# Dose-dependent expression of claudin-5 is a modifying factor in schizophrenia

**DOI:** 10.1038/mp.2017.156

**Published:** 2017-10-10

**Authors:** C Greene, J Kealy, M M Humphries, Y Gong, J Hou, N Hudson, L M Cassidy, R Martiniano, V Shashi, S R Hooper, G A Grant, P F Kenna, K Norris, C K Callaghan, M dN Islam, S M O’Mara, Z Najda, S G Campbell, J S Pachter, J Thomas, N M Williams, P Humphries, K C Murphy, M Campbell

**Affiliations:** 1 Department of Genetics, Smurfit Institute of Genetics, Lincoln Place Gate, Trinity College Dublin, Dublin, Ireland; 2 Division of Renal Diseases, Department of Internal Medicine, Washington University School of Medicine, St Louis, MO USA; 3 Department of Pediatrics, Duke University Medical Center, Durham, NC USA; 4 Department of Allied Health Sciences, University of North Carolina School of Medicine, Chapel Hill, NC USA; 5 Department of Neurosurgery, Stanford University School of Medicine, Stanford, CA USA; 6 Biosciences Department, Faculty of Health and Wellbeing, Biosciences and Chemistry, Sheffield Hallam University, Sheffield, UK; 7 Trinity College Institute of Neuroscience, Trinity College Dublin, Dublin, Ireland; 8 School of Psychology, Trinity College Dublin, Dublin, Ireland; 9 Department of Cell Biology, University of Connecticut Health Center, Farmington, CT USA; 10 Department of Psychological Medicine and Neurology, MRC Centre in Neuropsychiatric Genetics and Genomics, Cardiff University School of Medicine, Cardiff, UK; 11 Department of Psychiatry, Royal College of Surgeons in Ireland, Dublin, Ireland

## Abstract

Schizophrenia is a neurodevelopmental disorder that affects up to 1% of the general population. Various genes show associations with schizophrenia and a very weak nominal association with the tight junction protein, claudin-5, has previously been identified. Claudin-5 is expressed in endothelial cells forming part of the blood-brain barrier (BBB). Furthermore, schizophrenia occurs in 30% of individuals with 22q11 deletion syndrome (22q11DS), a population who are haploinsufficient for the *claudin-5* gene. Here, we show that a variant in the *claudin-5* gene is weakly associated with schizophrenia in 22q11DS, leading to 75% less claudin-5 being expressed in endothelial cells. We also show that targeted adeno-associated virus-mediated suppression of claudin-5 in the mouse brain results in localized BBB disruption and behavioural changes. Using an inducible ‘knockdown’ mouse model, we further link claudin-5 suppression with psychosis through a distinct behavioural phenotype showing impairments in learning and memory, anxiety-like behaviour and sensorimotor gating. In addition, these animals develop seizures and die after 3–4 weeks of claudin-5 suppression, reinforcing the crucial role of claudin-5 in normal neurological function. Finally, we show that anti-psychotic medications dose-dependently increase claudin-5 expression *in vitro* and *in vivo* while aberrant, discontinuous expression of claudin−5 in the brains of schizophrenic patients post mortem was observed compared to age-matched controls. Together, these data suggest that BBB disruption may be a modifying factor in the development of schizophrenia and that drugs directly targeting the BBB may offer new therapeutic opportunities for treating this disorder.

## Introduction

Schizophrenia is a brain disorder that affects ~1% of the population and ~1.5 million people are newly diagnosed each year globally.^1^ It is characterized by delusions (fixed and false beliefs), hallucinations (visual and auditory), and disorganized thinking and speech that begin in early adulthood and continues through life.^2^ Symptoms can lead to abnormal social behaviours, depression and anxiety, and may lead to the 10% incidence of suicide in patients suffering from the condition.^3,4^ Little is known about its cause, however, much attention has focused on the role of neurochemistry and aberrant neural connectivity in the brains of subjects.^5,6^

The disease has a strong genetic component to it, with twin studies suggesting up to 80% heritability of the condition. Many genetic studies have identified linkage to chromosome 22, suggesting this region harbours major susceptible loci for schizophrenia.^7,8,9^ Intriguingly, individuals with the chromosomal abnormality 22q11 deletion syndrome (22q11DS) have a 30-fold increased lifetime risk of developing schizophrenia and other neuropsychiatric-related conditions due to microdeletions at the chromosomal region 22q11.21.^10,11,12^ 22q11DS occurs in ~1 in 4000 live births. Patients with 22q11DS display a distinctive set of developmental defects that can include cardiac abnormalities, intellectual disabilities and distinctive craniofacial patterning.^13^ The condition is characterized genetically by microdeletions within chromosome 22 and these deletions can be up to 3 Mb in size, which can comprise up to 40 genes. The deletions occur in only one copy of the 22q11 region of chromosome 22, leaving individuals essentially haploinsufficient for the genes within that region. Functional genetic studies associated with 22q11DS patients have previously focused on elucidating the role of individual genes within the deleted region in the hope that genetic predisposition to schizophrenia can be identified. One major component of the so-called blood-brain barrier (BBB), the gene *claudin-5*, is located within chromosome 22q11.21.

Of the numerous biological barriers throughout the body, the BBB is one of a few highly selective and tightly regulated barriers, reflecting the brain’s critical roles in cognitive function.^14^ The BBB is essential in regulating the exchange of ions and nutrients between the blood and brain and vice versa, while also protecting delicate neural tissue from potentially damaging blood-borne agents such as pathogens, immune cells and anaphylatoxins. Owing to this specialized barrier, central nervous system endothelial cells are distinct from endothelial cells of the periphery in several ways. Specifically, BBB-enriched transporter proteins control the entry and exit of metabolites across cells (transcellular pathway). In addition, highly electrical-resistant tight junctions (TJs) limit the flux between adjacent endothelial cells (paracellular pathway) and an absence of fenestrations (pores to allow the rapid exchange of molecules between blood and tissue in peripheral endothelial cells) function to limit the movement of molecules.^15,16,17,18^

Previous studies examining systemic biomarkers of BBB dysfunction (in particular, S-100β) suggest the BBB may be compromised in patients with schizophrenia,^19,20^ however, this is an indirect correlation of BBB dysfunction, and a distinct molecular genetic association between BBB dysfunction and schizophrenia has not been described previously. Moreover, the BBB is not a static microenvironment, it is highly dynamic in both homoeostatic physiology and pathology.^21,22^

Here, we describe the involvement of the most enriched TJ protein at the BBB, claudin-5, in predisposing an increased risk of schizophrenia in individuals with 22q11DS. In addition, we show that persistent and targeted suppression of claudin-5 at the BBB in mouse models induces a correlative phenotype of psychosis-like behaviours suggesting a regulation of BBB integrity and dynamism may be therapeutically relevant for the treatment of schizophrenia.

## Materials and methods

### Animal experiments and experimental groups

All studies carried out in the Smurfit Institute of Genetics in Trinity College Dublin (TCD) adhere to the principles laid out by the internal ethics committee at TCD and all relevant national licences were obtained prior to commencement of all studies. All mice were bred on-site in the specific pathogen-free unit at the Smurfit Institute of Genetics in TCD.

### Genotyping of patients for SNP rs10314

Ethical approval for human studies was obtained through the Royal Victoria Eye and Ear Hospital. Informed consent was obtained from all subjects. DNA of 100 ng from patients was amplified by PCR in a volume of 50 μl using 1 × reaction buffer, 200 μm each of dNTPs, 0.2 μm of forward and reverse primers, and 1.25 units of DNA Taq polymerase under the following conditions: 95 °C 5 min; (95 °C 1 min; 58 °C 1 min; 72 °C 1 min) × 34; 72 °C 5 min; 4 °C hold. This produced an amplified product of 603 bp using the following primers: forward primer 5′--3′ and reverse primer 5′--3′. The amplified product was digested with restriction endonuclease PvuII giving fragments of 177+199+227 and 199+404 for the g and c alleles, respectively. DNA from the above amplification was purified using a QIAquick PCR purification kit (Qiagen, Manchester, UK) and subjected to direct sequencing using the forward primer (above).

### Construction of claudin-5 pcDNA3-EGFP

Two human claudin-5 complementary DNAs (cDNAs) with 3′-UTRs containing the g and c alleles of Snp rs10314, respectively, were synthesized by GeneArt (Dublin, Ireland) and subcloned into the HindIII/XhoI site of pcDNA3-EGFP (Addgene, Cambridge, MA, USA).

### Polysome analysis

Hek293 were grown to 70% confluency, and polysome extracts were prepared as previously described^23^ with the following modifications, 0.5% (v/v) Triton X-100 and 1 mg/ml RNasin was added to the lysis buffer. Cyclohexamide (CHX) of 100 μg/ml was added to cells and then incubated on ice water for 30 min. Cells were subsequently scraped into 10 ml PBS and 100 μg/ml CHX. The cells were pelleted at 400 *g* for 4 min and then resuspended in lysis buffer. Following this, the cells were lysed further with a 25 gauge needle and left on ice for 10 min. Extracts were layered onto 15–50% sucrose gradients. The gradients were sedimented via centrifugation at 40 000 r.p.m. in a Beckman (Clare, Ireland) ultracentrifuge for 2.5 h, and the *A*_254_ was measured continuously to give the traces shown. Fourteen fractions were collected across the gradient into two volumes of trizol (Life Technologies, Dublin, Ireland), and the RNA was extracted, precipitated and resuspended in diethyl-pyrocarbonate-treated water. cDNA was prepared and real-time reverse transcription polymerase chain reaction (RT-PCR) was carried out; from this, claudin-5 transcript levels in each fraction were determined as a percentage of total RNA.

### Real-time RT-PCR analysis

Transcript levels were quantified using a two-step RT-PCR on the 7300 real-time PCR System (Applied Biosystems, Dublin, Ireland) with QuantiTect SYBR Green I (Qiagen) as a fluorescent dye. cDNA was reverse-transcribed from RNA with the high-capacity cDNA reverse transcription Kit (Applied Biosystems). Real-time PCR was performed with the FastStart Universal SYBR Green Master (ROX) master mix (Roche, Dublin, Ireland). The RT-PCR reaction conditions were as follows: 95 °C × 10 min, (95 °C × 10 s, 60 °C × 30 s) × 40, 95 °C × 15 s, 60 °C × 1 min, 95 °C × 15 s, 60 °C × 15 s. The primer sequences for the RT-PCR experiments were supplied by Sigma-Aldrich (Wicklow, Ireland) and were as follows: claudin-5 left, 5′--3′, and right, 5′--3′; β-actin left, 5′--3′ and right, 5′--3′. Relative gene expression levels were measured using the comparative *C*_T_ method (ΔΔ*C*_T_). Expression levels of target genes were normalized to the housekeeping gene β-actin.

### Cell culture and transfection

Human embryonic kidney cells (HEK293) were cultured in Dulbecco’s modified Eagle’s medium supplemented with 10% foetal calf serum in a 5% CO_2_ incubator at 37 °C. One day before transfection, HEK293 cells were seeded on 12-well plates (2.5 × 10^5^ cells per well). The next day, 500 ng of plasmid containing wild-type or rs10314 claudin-5 cDNA was transfected per well using Lipofectamine 2000 (Invitrogen, Dublin, Ireland). Mouse brain endothelial cells (Bend.3, American Type Culture Collection, Manassas, VA, USA) were cultured in Dulbecco’s modified Eagle’s medium supplemented with 10% foetal calf serum and 2 mm sodium pyruvate in a 5% CO_2_ incubator at 37 °C. Bend.3 cells were seeded on 12-well plates (2.5 × 10^5^ cells per well) and 100 ng/ml claudin-5 short hairpin RNA (shRNA) was transfected per well using Lipofectamine 2000. RNA was extracted from HEK293 cells and Bend.3 cells with the E.Z.N.A Total RNA Kit 1 (Omega biotek, Norcross, GA, USA) according to the manufacturer’s instructions. Proteins were isolated with lysis buffer (62.5 mm Tris, 2% SDS, 10 mm dithiothreitol, 10 μl protease inhibitor cocktail/100 ml (Sigma-Aldrich), followed by centrifugation at 12 000 r.p.m. for 20 min at 4 °C and supernatant was removed for claudin-5 protein analysis.

### Adeno-associated virus production

shRNAs designed to target transcripts derived from mouse claudin-5 were incorporated into adeno-associated virus (AAV)-2/9 vectors. shRNA was cloned into the pSingle-tTS-shRNA (Clontech, Mountain View, CA, USA) vector. The plasmid incorporating the inducible system with claudin-5 shRNA was digested with BsrBi and BsrGI, and ligated into the Not1 site of the plasmid pAAV-MCS, such as to incorporate left and right AAV-inverted terminal repeats. (L-ITR and R-ITR). AAV-2/9 was then generated using a triple transfection system in a stably transfected HEK-293 cell line for the generation of high-titre viruses (Vector BioLabs, Malvern, PA, USA).

### Injection of AAV for claudin-5 suppression in the hippocampus and medial prefrontal cortex

C57/BL6J mice (8–12 weeks old) were anaesthetized using a ketamine/metadomidine mixture administered via intraperitoneal injection and placed in a stereotaxic frame. An incision was made to expose the skull, and burr holes were made using a surgical drill either above the dorsal hippocampus or the medial prefrontal cortex (mPFC). A Hamilton syringe was loaded with an AAV expressing either a shRNA against claudin-5 or a non-targeting (NT) control, and the needle was slowly lowered into the dorsal hippocampus: (co-ordinates: A/P=−1.9 mm; M/L=±1.55 mm; D/V=1.75 mm, or mPFC: (co-ordinates: A/P=+1.9 mm; M/l=±0.4 mm; D/V=2.5 mm). AAV solution of 2.0 μl was then injected at a rate of 0.5 μl per min, and once complete, the needle was left in place for 5 min before repeating the procedure in the other hemisphere. Anaesthesia was reversed with an intraperitoneal injection of atipamezole and placed in an incubator until recovered. All mice were given 7 days of recovery before introducing doxycycline into their drinking water (2 mg/ml in 2% sucrose solution). Doxycycline treatment was continued for the length of the behavioural experiments, with behavioural testing beginning 14 days after the introduction of doxycycline to drinking water to ensure maximal suppression of claudin-5. Behavioural testing was then performed for ~4 weeks before animals were killed for histological and molecular analysis. In addition to mice used for behavioural experiments (see below), a small cohort were injected with an AAV-expressing green fluorescent protein to visualize the extent of AAV localization following injection.

### Immunocytochemistry and immunohistochemistry

HEK293 cells and Bend.3 cells were seeded on 1% fibronectin-coated Nunc Lab-Tek II Chamber Slides (Thermo Scientific, Dublin, Ireland) in Dulbecco’s modified Eagle’s medium. After plasmid or shRNA transfection, cells were fixed for 10 min at room temperature with ice-cold methanol, washed twice with PBS and incubated with 5% normal goat serum before overnight incubation with polyclonal rabbit anti-claudin-5 (1:100). Cells were then washed twice with PBS and incubated with Cy3-conjugated goat anti-rabbit IgG secondary antibody (1:500; Abcam, Cambridge, UK) for 2 h at room temperature and counterstained with Hoechst 33258 to visualize nuclei.

Mice were killed and the brains quickly removed. One hemisphere was embedded in optimal cutting temperature compound (VWR, Dublin, Ireland), snap-frozen in liquid nitrogen and stored at −20 °C prior to slicing on a cryostat. Mouse brain cryosections (12 μm thick) were post-fixed in ice-cold methanol for 10 min at room temperature and washed three times in PBS. Sections were then incubated with 5% normal goat serum before overnight incubation with primary antibodies (rabbit anti-claudin-5 1:100, rabbit anti-ZO-1 1:100 and rabbit anti-occludin 1:100, Life Technologies). Sections were double-stained with isolectin-IB4-Alexa Fluor 488 1:300, Life Technologies, to label vessels. For permeability experiments, sections were incubated with Fluorescein isothiocyanate-conjugated polyclonal rabbit anti-human fibrinogen 1:100, DAKO, or Cy3-conjugated streptavidin 1:100, Sigma-Aldrich, overnight at 4 °C. Following three washes in PBS, sections were incubated with Cy3-conjugated goat anti-rabbit IgG secondary antibody (1:500; Abcam) for 2 h at room temperature, washed three times with PBS and counterstained with Hoechst 33258 for 30 s at a dilution of 1:10 000 of a stock 1 mg/ml solution to visualize nuclei. Sections were mounted and coverslipped with Aqua Poly/mount (Polysciences, Washington, PA, USA). Sections were imaged with a Zeiss LSM 710 confocal laser scanning microscope (Cambridge, MA, USA). TJ signal intensity was quantified with the region of interest defined by isolectin-IB4 staining. For biotin and fibrinogen quantification, the average signal intensity of 10 regions of interest outside the blood vessels was normalized to the average signal intensity of 10 regions of interest within the blood vessels. All image analysis was performed in ImageJ (National Institutes of Health, Rockville, MD, USA).

### Behavioural analyses

For detailed methodology for behavioural analyses, please see Supplementary Methods.

### Primary mouse brain microvascular endothelial cell isolation

Microvessels were isolated from cortical grey matter of C57BL/6J mice by collagenase/dispase (Roche) digestion and bovine serum albumin density gradient centrifugation. Purified vessels were seeded onto collagen IV/fibronectin-coated tissue-culture plates or Corning (Corning, NY, USA) HTS 24-well Transwell polyester inserts (0.4 μm pore size, vessels from five mouse brains per 3 ml) at high density. Cells were grown in EGM2-MV (Lonza, Cambridge, UK) (with 5 μg/ml puromycin during the first 3 days for endothelial cell selection) for 2–3 weeks until their transendothelial electrical resistance values plateaued.

### Drug treatments

Haloperidol, lithium chloride and chlorpromazine were purchased from Sigma-Aldrich. Confluent primary mBMEC cells were treated with 0.2, 2 and 20 μm chlorpromazine, or haloperidol diluted in culture medium containing 0.1% DMSO, and 0.1, 1 and 10 mm lithium diluted in culture medium. For *in vivo* injections, haloperidol and chlorpromazine were diluted in 2.5% polyethylene glycol 400 in saline and 200 μl was injected via tail vein at a dose of 1 mg/kg body weight. Lithium was diluted in saline and injected at a dose of 100 mg/kg body weight. Animals were killed 24 h following injection, and tissues were processed for protein and RNA analysis.

### Generation of inducible claudin-5 knockdown mice

Mice were generated by standard blastocyst injection^24^ at Charles Rivers Laboratory (Mayo, Ireland) using their strict VAF/EliteTM health standards. A doxycyline -inducible claudin-5 shRNA (160 variant) was inserted at the Col1a1 locus on chromosome 11. In addition, a CAG-lox-stop-lox-rtTA3-IRESmKate2 (CLR3K) allele was knocked in at the endogenous *Rosa26* locus on chromosome 6. This gene utilizes the endogenous *Rosa26* promoter to drive expression of the reverse tetracycline-controlled transactivator (M2rtTA), once mice are crossed to a Cre-recombinase expressing mouse.^25,26,27^ Mice were maintained as homozygous for the claudin-5 shRNA-containing gene and the *rtTA* gene. When required for experiments, mice were crossed to transgenic Tie-2-Cre expressing animals and Cre-negative mice were used as littermate controls.

For behavioural experiments, all mice (Cre-positive and Cre-negative) were given doxycycline in their drinking water (2 mg/ml in 2% sucrose solution) and kept on doxycycline for the length of the behavioural experiments. Behavioural testing began 14 days after the introduction of doxycycline to drinking water to ensure maximal suppression of claudin-5. Behavioural testing was then performed for ~4 weeks before animals were killed for histological and molecular analysis.

### Magnetic resonance imaging

BBB integrity was assessed *in vivo* via magnetic resonance imaging (MRI), using a dedicated small rodent 7 T MRI system located at TCD (www.neuroscience.tcd.ie/technologies/mri.php). Anaesthetized mice were physiologically monitored (electrocardiogram, respiration and temperature) and placed on an MRI-compatible support cradle, with a built-in system for maintaining the animal’s body temperature at 37 °C. The cradle was then positioned within the MRI scanner. Accurate positioning was ensured by acquiring an initial rapid pilot image, which was then used to ensure the correct geometry was scanned in all subsequent MRI experiments. Upon insertion into the MRI scanner, high-resolution anatomical images of the brain were acquired (100 μm in-plane and 500 μm through-plane spatial resolution). To visualize brain damage and lesion volumes, high-resolution images were acquired using Rapid Acquisition with Relaxation Enhancement (RARE) 2-D sequence with a RARE factor of 8 and an echo time resulting in an effective time of 42.2 ms (with a flip angle of 180). With an acquisition matrix of 128 × 128 and a field of view of 1.8 × 1.8 cm^2^, the pixel resolution was 0.141 mm per pixel. In the coronal plane, 15 slices, each measuring 0.25 mm in thickness were acquired. Repetition time was 7274.2 ms, and four averages were used for a total measuring time of 7 min 45 s.

Compromises of the BBB were then visualized in high-resolution T1-weighted MR images (resolution, 0.156 × 0.156 × 5 mm^3^; field of view: 20 × 20 × 17.9 mm^3^; matrix: 128 × 128 × 30; TR/TE: 500/2.7 ms; flip angle: 30; number of averages: 3; acquisition time: 2 min, 24 s; repetitions: 12), following administration of 100 μl of a 1 in 3 dilution of Gd-DTPA (gadolinium diethylene-triamine penta-acetic acid), administered via the tail vein. Image analysis was performed in ImageJ, where regions of interest were drawn and signal intensity changes were measured as mean pixel intensity and normalized to the signal intensity prior to injection of Gd-DTPA.

### Electroretinography analysis of mice

Cre-positive Cl5-160 mice were dark-adapted overnight and prepared for electroretinography under dim red light. Pupillary dilation was carried out by instillation of 1% cyclopentalate and 2.5% phenylephrine. Animals were anesthetized by intraperitoneal (i.p.) injection of ketamine (2.08 mg per 15 g body weight) and xylazine (0.21 mg per 15 g body weight). Standardized flashes of light were presented to the mouse in a Ganzfeld bowl to ensure uniform retinal illumination. The electroretinography responses were recorded simultaneously from both eyes by means of gold wire electrodes (Roland Consult, Brandenburg, Germany) using Vidisic (Dr Mann Pharma, Berlin, Germany) as a conducting agent and to maintain corneal hydration.

### Acoustic prepulse inhibition

Sensorimotor gating was assessed by prepulse inhibition (PPI) of the acoustic startle response. The PPI apparatus consisted of a soundproof PPI chamber with a weighing scale positioned in the centre of the chamber beside a loudspeaker. Mice were maintained in a holding chamber placed on the scale. Each mouse was given 2 days to habituate to the holding chamber and PPI chamber with a constant background white noise (65 dB). Before the beginning of the experiment, all instruments were calibrated and startle stimulus (71, 77, 83, 100, 110, 120 dB) were set. PPI was divided into three stages:

1. Two minutes habituation with constant background noise of 65 dB.
2. Presentation of prepulse (71, 77, 83 dB) and pulse (100, 110, 120 dB) intensities, to habituate animals to startle stimulus.
3. Ten blocks of random combinations of prepulse alone, pulse alone, prepulse plus pulse and no stimulus trials.

Startle stimulus were presented as 20 ms bursts of white noise with an interstimulus (time between presentation of prepulse and pulse stimuli) interval of 100 ms. Data were averaged across the 10 blocks for each prepulse, pulse, prepulse plus pulse and no stimulus trial, and PPI was calculated as:

%PPI = 100 × (pulse−prepulse plus pulse)/pulse.

### Human brain sections

Free-floating 60 μm-thick sections of post-mortem human brain tissue from 24 deceased schizophrenia patients and 24 age-matched controls were obtained from the Stanley Medical Research Institute. In addition to information relating to their schizophrenia diagnosis, the data accompanying these samples also included genotype information. Sections were washed in PBS and antigen retrieval was performed by boiling the sections for 2 × 5 min in sodium citrate buffer (10 mm sodium citrate, 0.05% Tween 20, pH 6.0). Sections were permeabilized with 0.5% Triton X-100; blocked with 5% NGS; and incubated with monoclonal mouse anti-claudin-5 (1:50; Santa Cruz, Dallas, TX, USA) for 48 h at 4 °C. Brain sections were incubated with Cy3-conjugated goat anti-mouse IgG (1:300, Abcam) for 3 h at room temperature and counterstained with Hoechst 33258. *Z*-stacks were acquired with identical acquisition settings across samples and three-dimensional images were rendered with ImageJ 3D viewer. Staining and microscopy were performed blind to diagnosis. Quantification of claudin-5 staining (intensity and continuity) in these samples was performed using software designed for assessing blood vessel networks (Angiogenesis Analyser plug-in for ImageJ, National Institutes of Health). Briefly, the red channel (claudin-5) from the confocal images (at × 10 magnification) was isolated and was processed to reduce background staining. The software was then set to map the blood vessels present in each section and the total length of the vessels was quantified (number of pixels). This quantified data was analysed based on diagnosis and genotype.

### Statistical analyses

Statistical analysis was performed using Student’s *t*-test, with significance represented by a *P*-value of ⩽0.05. For multiple comparisons, analysis of variance was used with a Tukey–Kramer post-test and significance represented by a *P*-value of ⩽0.05. G*Power was used to choose sample size and ensure adequate power for experiments.

## Results

### Claudin-5 variant rs10314 weakly associates with schizophrenia in 22q11DS patients and causes decreased protein expression

**Figure 1:**
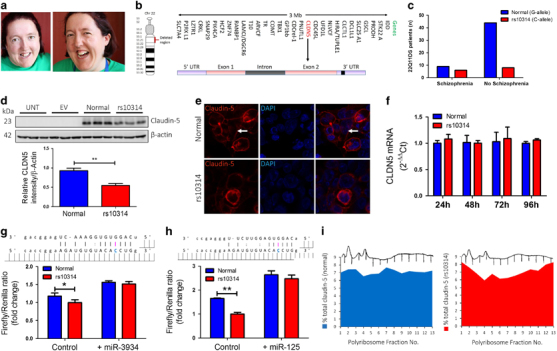
Claudin-5 variant rs10314 causes decreased protein expression. (**a**) Distinctive craniofacial pattern characteristic of 22q11DS. (**b**) Chromosomal location of *claudin-5* gene on Chr. 22q11.21. (**c**) Weak association of the claudin-5 variant rs10314 in a population of 67 22q11DS patients (*P=0.0388, two-sided *χ* ^2^-test). (**d**) Claudin-5 expression in HEK-293 cells expressing normal or rs10314 variant claudin-5. Claudin-5 protein is significantly decreased in rs10314-expressing plasmid 24 h post transfection (**P<0.01). (**e**) Claudin-5 expression in HEK-293 cells expressing wild-type (white arrows) or rs10314-expressing cDNA. (**f**) Levels of claudin-5 transcript remain unchanged 24, 48, 72 and 96 h post transfection of wild-type and rs10314-expressing cDNA. (**g**) Analysis of hsa-MiR-3934 and (**h**) hsa-MiR-125a-3p binding within 3′-untranslated region of the *claudin-5* gene. The rs10314 variant shows reduced promoter activity compared to the normal variant in the control condition (*P<0.05; **P<0.01). This difference is absent following the addition of either miRNA. (**i**) Levels of expression of claudin-5 transcript in polysome fractions post transfection of normal (blue) or rs10314 (red)-expressing cDNA vectors. cDNA, complementary DNA; EV, empty vector; miRNA, micro RNA; UNT, untransfected.

Individuals with 22q11DS display a distinctive set of developmental defects that can include cardiac abnormalities, intellectual disabilities and distinctive craniofacial patterning (Figure 1a). The condition is characterized genetically by microdeletions within chromosome 22 leaving individuals essentially haploinsufficient for the genes within that region, and the critical BBB associated gene, claudin-5, is located here (Figure 1b). In 2004, it was reported that a single-nucleotide polymorphism in the 3′-UTR of the claudin-5 locus (rs10314) was weakly associated with schizophrenia in a Han Chinese population.^28^ This weak nominal association was also observed in other studies.^29,30,31^ Following sequencing across the remaining claudin-5 allele from a cohort of 67 22q11DS patients, a weak nominal association (*P=0.0388, two-sided *χ* ^2^-test) between the claudin-5 variant (rs10314) and those 22q11DS patients who went on to develop schizophrenia was observed (Figure 1c).

Highest frequencies for the derived allele at rs10314 in modern populations (1000 Genomes Project Consortium) are observed to occur in Eastern Eurasia and Siberia (~31%), with notable peaks occurring in Northeast Siberian populations such as the Even, Evenki, Koryak and Yakut (42–67%) (Supplementary Figures 1 and 2 and Supplementary Table 1), with lowest frequencies observed in Afro-Asiatic speaking groups of Western Asia (3.23%).

Generation of cDNA-expressing vectors of normal and rs10314 variant *claudin-5* genes showed the rs10314 variant gene expresses 50% less claudin-5 protein in cells (Figure 1d), suggesting that individuals with 22q11DS carrying the rs10314 variant may express up to 75% less claudin-5 at their BBB than the general population (50% for 22q11DS with the wild-type variant due to haploinsufficiency and down by 75% for 22q11DS with the rs10314 variant on top of haploinsufficiency). Interestingly, the variant claudin-5 was less evident at the plasma membrane of transfected cells, with positive immunoreactivity observed in perinuclear bodies, possibly lysosomes (Figure 1e). There was however no difference in the levels of claudin-5 transcript over 96 h (Figure 1f), suggesting this variant confers a change in the post-translational processing or stability of the protein product. Transfection of the normal or variant constructs in cells with well-established TJs (Caco-2 cells) showed the same decrease in claudin-5 expression (Supplementary Figure 3).

The rs10314 SNP is a G to a C base change in the 3′-UTR of the *claudin-5* gene. In this regard, we sought to examine if micro RNA (miRNA)-binding sites were impacted in any way. In the absence of miR-3934 or miR-125a-3p, two miRNAs in which that variant confers novel binding, the normal variant expresses significantly more promoter activity compared to the rs10314 variant (*P<0.05 and **P<0.01 for the respective miRNAs). However, in the presence of either miRNA, there were no significant differences in promoter binding between the normal and rs10314 variants (Figures 1g and h). Isolation of polysome fractions however showed a distinct shift in the levels of claudin-5 transcript in the rs10314 variant to the sub-polysome fractions, suggesting efficient translation is being impacted upon (Figure 1i).

### Site-specific and chronic suppression of claudin-5 induces distinct behavioural changes in mice

**Figure 2:**
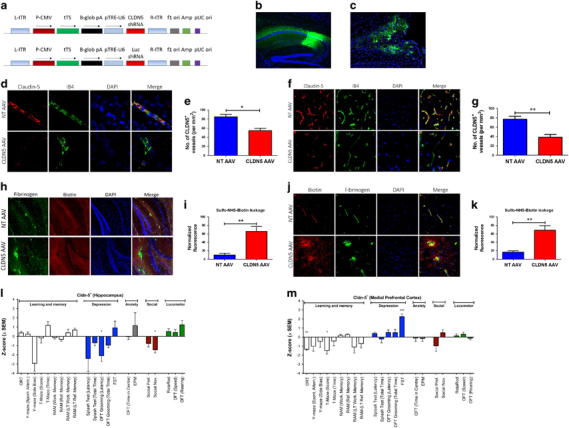
Site-specific suppression of claudin-5 in the hippocampus and medial prefrontal cortex. (**a**) Plasmid maps of claudin-5 AAV-2/9 and non-targeting (NT) AAV-2/9. (**b**) eGFP-expressing AAV-2/9 injected into the dorsal hippocampus. (**c**) eGFP-expressing AAV-2/9 injected into the medial prefrontal cortex (mPFC). (**d** and **e**) Significant suppression of claudin-5 (red) in the microvasculature of the hippocampus (IB4: green, DAPI: blue; scale bar: 50 μm; *P<0.05). (**f** and **g**) Significant suppression of claudin-5 (red) in the microvasculature of the mPFC (IB4: green, DAPI: blue; scale bar: 50 μm; **P<0.01). (**h**) Biotin (red) and fibrinogen (green) extravasation in the hippocampus; scale bar: 50 μm. (**i**) Quantification of biotin extravasation in the hippocampus, suppression of claudin-5 significantly increases the amount of extravasation (**P<0.01). (**j**) Biotin (red) and fibrinogen (green) extravasation in the mPFC; scale bar: 50 μm. (**k**) Quantification of biotin extravasation in the mPFC, suppression of claudin-5 significantly increases the amount of biotin extravasation (**P<0.01). (**l**) Summary of behavioural data following suppression of claudin-5 in the hippocampus. These mice showed significantly decreased levels of grooming (*P<0.05) along with a significant impairment in the social novelty task (*P<0.05). (**m**) Summary of behavioural data following suppression of claudin-5 in the mPFC. These mice showed significant impairments in the social object recognition task (**P<0.01) and the T-maze (*P<0.05) along with a significant enhancement in the forced swim test (***P<0.001). Behavioural assays were performed 2 weeks post supplementation of doxycycline (2 mg/ml) to the drinking water. AAV, adeno-associated virus; eGFP, enhanced green fluorescent protein; EPM, elevated plus maze; FST, forced swimming test; LT, long-term; shRNA, short hairpin RNA; RAM, radial arm maze; OFT, open field test.

Such is the impact of the BBB on neural integrity, it can be suggested that each neuron is essentially perfused by its own capillary. We generated inducible AAV vectors expressing doxycycline-inducible claudin-5 shRNA to allow for targeted suppression of claudin-5 in the brains of mice (Figure 2a). We used AAV-2/9 that allows for transduction of endothelial cells within a region of tissue of ~1 mm^3^ (Supplementary Figure 4; for further details on claudin-5 suppression in brain tissue using this method, see ref.^32^). Mice were injected bilaterally in the hippocampus (Figure 2b) or mPFC (Figure 2c) with AAV-expressing claudin-5 shRNA or a NT AAV as outlined. Claudin-5 levels were significantly suppressed following supplementation of doxycycline in the water of mice both in the hippocampus (*P<0.05, Figures 2d and e) and mPFC (**P<0.01, Figures 2f and g). Claudin-5 suppression did not trigger vascular remodelling events as measured by the total stained length of isolectin-IB4 vessels in NT or claudin-5 AAV-injected groups (Supplementary Figure 4c). Suppression of claudin-5 in the hippocampus or mPFC had no impact on the expression levels of occludin or ZO-1 (Supplementary Figure 5). There was also a significant increase in extravasation of a biotinylated agent (600 Da) and fibrinogen (340 kDa) in both the hippocampus (**P<0.01, Figures 2h and i) and mPFC (**P<0.01, Figures 2j and k).

Intriguingly, a range of behavioural changes were observed in mice across five domains (learning and memory; depression; anxiety; social behaviour; and locomotor activity). Suppression of claudin-5 in the hippocampus led to a significant impairment in performance in the social novelty task (*P<0.05) and a significant decrease in grooming behaviour (*P<0.05), suggestive of changes in affect (Figure 2l). Suppression of claudin-5 in the mPFC led to more profound changes, with significant impairments in recognition memory (**P<0.01) and working spatial memory (*P<0.05) in addition to non-significant impairments in performance in the Y-maze and radial arm maze (Figure 2m). In addition, claudin-5 suppression in the mPFC significantly enhanced performance in the forced swim test (***P<0.001), with mice spending less time immobile compared to controls. Given the lack of hyperlocomotion measured on other tests, this is suggestive of increased resilience to the stress associated with the task. More detailed readouts from these experiments are outlined in Supplementary Figure 6.

### Generation and characterization of inducible claudin-5 knockdown mice

**Figure 3:**
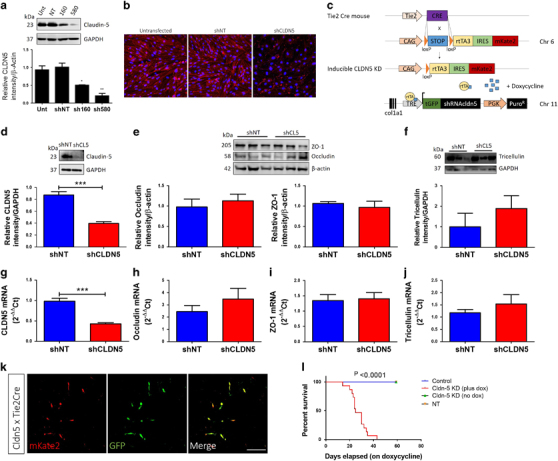
Generation and characterization of inducible claudin-5 knockdown mice. (**a**) Significant suppression of claudin-5 expression 24 h post transfection of two different shRNAs targeting claudin-5 *in vitro* (*P<0.05; **P<0.01). (**b**) Levels of expression of claudin-5 at the tight junction 24 h post transfection of claudin-5 shRNA in a monolayer of mouse brain endothelial cells; scale bar: 50 μm. (**c**) Schematic representation of inducible claudin-5 knockdown mouse model. (**d**) Inducible suppression of claudin-5 protein using shRNA in the vasculature of the mouse brain (***P<0.001) 72 h following i.p. injection of 40 mg/kg doxycycline in 0.9% saline. No significant changes in the protein expression levels of the other tight junction proteins, (**e**) occludin, and ZO-1 and (**f**) tricellulin. (**g**) Inducible suppression of claudin-5 mRNA using shRNA in the vasculature of the mouse brain (***P<0.001). No changes in the transcript levels of (**h**) occludin, (**i**) ZO-1 or (**j**) tricellulin. (**k**) Expression of rtTA3 (red, mKate) and claudin-5 shRNA (green, turbo green fluorescent protein) confirmed in the vasculature of the mouse brain 72 h following i.p. injection of doxycycline; scale bar: 50 μm. (**l**) Survival chart for inducible claudin-5 knockdown mice. Inducible claudin-5 knockdown mice supplemented with doxycycline (2 mg/ml) all die compared to inducible claudin-5 mice fed water only, a scrambled control mouse (NT) and Cre-negative littermates. mRNA, messenger RNA; NT, non-targeting; shRNA, short hairpin RNA.

As claudin-5 knockout mice die within hours of birth,^33^ we generated a mouse model that allows for inducible RNAi-based suppression of claudin-5 levels at the BBB. We first identified two shRNAs, 160 and 580, that would sufficiently suppress transcript and protein levels of claudin-5 *in vitro* versus untreated and NT controls (Figures 3a and b). Subsequently, we generated mice such that a gene containing a doxycycline-inducible claudin-5 160 shRNA was inserted at the Col1a1 locus on chromosome 11. In addition, a CAG-lox-stop-lox-rtTA3-IRESmKate2 (CLR3K) allele was knocked in at the endogenous *Rosa26* locus on chromosome 6. This gene utilizes the endogenous *Rosa26* promoter to drive expression of the reverse tetracycline-controlled transactivator (M2rtTA), once mice are crossed to a Cre-recombinase expressing mouse.^25,26,27^ When we cross the bi-allelic mice outlined above to Tie-2-Cre mice (Figure 3c), animals that are Cre-positive show significantly decreased levels of claudin-5 protein in their brain when administered doxycycline in their diet (***P<0.001; Figure 3d) in addition to significantly lower levels of claudin-5 messenger RNA (***P<0.001). In addition, using mKate (red) and green fluorescent protein (green) reporter genes, confirmation of rtTA and claudin-5 shRNA expression specifically in endothelial cells was assessed. Suppression of claudin-5 did not significantly impact on the levels of expression of other TJ components such as ZO-1, occludin or tricellulin, either in the amount of translated protein or at the transcript level (Figures 3h–j). Finally, suppression of claudin-5 had a significant impact on the animals’ lifespan; persistent suppression of claudin-5 in adult mice led to mortality across all animals in this study within 40 days, unlike Cre-negative littermate controls, inducible claudin-5 mice housed with normal drinking water, and mice expressing a scrambled NT shRNA on doxycycline (Figure 3k). This persistent suppression of claudin-5 also induced very distinct phenotypic readouts in the 48 h prior to death, including behavioural arrest, tail flicking and hyperlocomotion (Supplementary Videos 1 and 2).

### Inducible claudin-5 knockdown mice display characteristic features of schizophrenia

**Figure 4:**
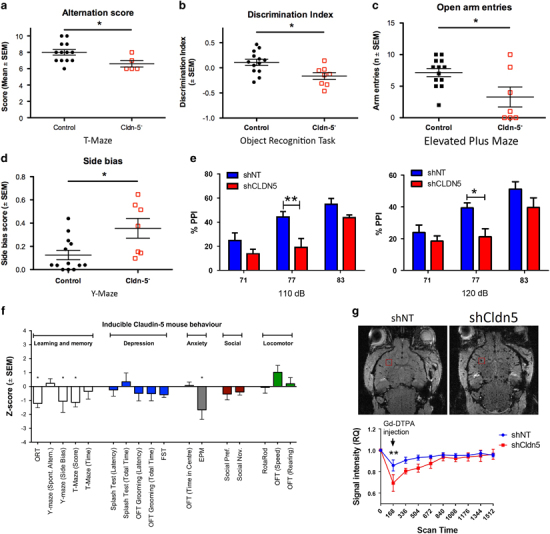
Phenotype of inducible claudin-5 knockdown mice. (**a**) Reduced spontaneous alternation in the T-maze in claudin-5 knockdown mice (*P<0.05). (**b**) Reduced discrimination index in claudin-5 knockdown mice in the object recognition task (*P<0.05). (**c**) Reduced open arm entries observed in the elevated plus maze in the claudin-5 knockdown mice (*P<0.05). (**d**) Increased side bias in inducible claudin-5 knockdown mice in a spontaneous alternation task in the Y-maze (*P<0.05). (**e**) Decreased acoustic prepulse inhibition (PPI) in inducible claudin-5 knockdown mice with a 77 dB prepulse at 110 dB (**P<0.01) and 120 dB (*P<0.05). (**f**) Summary of behavioural data following suppression of claudin-5 in the inducible claudin-5 knockdown mouse model. (**g**) Contrast-enhanced magnetic resonance imaging (MRI) showing significant extravasation of contrast agent in the brain of inducible claudin-5 knockdown mice (right) compared to non-targeting control mice (left; **P<0.01). All assays were performed 2–4 weeks post supplementation of doxycycline (2 mg/ml) to the drinking water. RQ, relative quantity.

Inducible knockdown mice showed significant impairments in learning and memory tasks, with performance in the T-maze (*P<0.05) and object recognition task (*P<0.05) particularly affected (Figures 4a and b). Performance in the spontaneous alternation task in the Y-maze was unimpaired due to the development of a significant side bias (*P<0.05; Figure 4d). These mice also displayed increased anxiety-like behaviour as assessed by a significant reduction in open arm entries in the elevated plus maze (*P<0.05; Figure 4c). While there were trends towards depression-like symptoms, decreased social activity and increased locomotion, none of these reached significance and are summarized in Figure 4f. Detailed readouts from these experiments are outlined in Supplementary Figure 12. Importantly, these mice did not have any visual deficits that may have impacted behaviour as examined by electroretinography measurements (Supplementary Figure 15).

Acoustic PPI is a sensorimotor gating phenomenon that is preserved across species and has previously been shown to strongly associate with schizophrenia. Indeed, numerous proposed mouse models of schizophrenia use acoustic PPI as a correlate of a schizophrenia-like phenotype. Here, we show that mice lacking sufficient claudin-5 at the BBB display a reduced acoustic PPI response with a 77 dB prepulse at 110 dB (**P<0.01) and 120 dB (*P<0.05; Figure 4e). Taken together, the behavioural data presented here suggest a profound link between the gene-dosage effect of claudin-5 and manifestations of many schizophrenia-associated symptoms.

Finally, *in vivo* measurements of BBB permeability were performed using contrast-enhanced MRI. In claudin-5-suppressed animals, gadolinium extravasation lead to significantly increased contrast in the acquired images (**P<0.01) compared to NT control mice (Figure 4g). In addition, there was significantly increased extravasation of fibrinogen throughout the brains of claudin-5 suppressed animals (**P<0.01) compared to littermate controls (Supplementary Figure 16).

### Anti-psychotic drugs regulate claudin-5 levels and claudin-5 expression is aberrant in human schizophrenia patients

**Figure 5:**
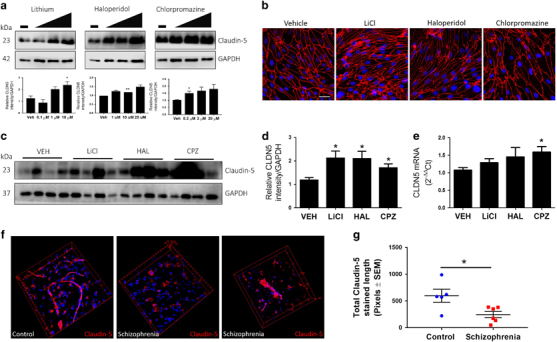
Regulation of claudin-5 levels by anti-psychotic drugs and examples of aberrant claudin-5 expression in schizophrenia. (**a**) Levels of expression of claudin-5 in primary mouse brain endothelial cells exposed for 24 h to lithium (LiCl), haloperidol (HAL) or chlorpromazine (CPZ). (**b**) Immunocytochemical analysis of claudin-5 (red)-staining pattern in primary mouse brain endothelial cells treated with anti-psychotic drugs; scale bar: 50 μm. (**c**) Levels of expression of claudin-5 in capillary fractions from mouse brains 24 h following administration (i.v.) of LiCl, HAL or CPZ. (**d**) Claudin-5 expression was significantly higher in mice treated with antipsychotics (*P<0.05). (**e**) Claudin-5 mRNA levels were significantly higher in mice treated with CPZ (*P<0.05). (**f**) Claudin-5 levels in normal control or schizophrenia donor brain tissues from the parietal lobe. Sixty-two per cent of schizophrenia patients showed aberrant claudin-5 staining in the parietal lobe. (**g**) There are significantly lower levels of claudin-5 in the parietal lobe in individuals who have a diagnosis of schizophrenia and the presence of the rs10314 allele compared to controls who have the rs10314 allele (*P<0.05).

It is known that systemic biomarkers of BBB dysfunction are increased in individuals with schizophrenia. Evidence for microvascular dysfunction has also previously been linked with the condition,^34^ however, a distinct molecular genetic link between endothelial cell dysfunction and schizophrenia has been lacking. We examined the effects of some of the most common anti-psychotic drugs on claudin-5 levels both *in vitro* in primary brain endothelial cell cultures and *in vivo* in wild-type c57/BL6 mice. We found that lithium, haloperidol and chlorpromazine all significantly increased levels of claudin-5 protein in a dose-dependent manner *in vitro* (Figure 5a) with no obvious changes in the expression pattern of claudin-5 (Figure 5b). *In vivo*, we found similar increases in claudin-5 following anti-psychotic administration with significant increases in protein expression for all drugs (*P<0.05; Figures 5c and d) and in messenger RNA transcription for chlorpromazine alone (*P<0.05; Figure 5e). We also examined the impact of these drugs on the levels of other TJ components, and found that lithium and haloperidol increased levels of occludin (*P<0.05) but not ZO-1 *in vitro* (Supplementary Figures 17a–c), while chlorpromazine alone increased levels of ZO-1 (*P<0.05) *in vivo* (Supplementary Figures 17d–f). To explore whether these drugs were affecting claudin-5 expression through Wnt signalling, we analysed downstream components of this signalling and found that levels of Axin-2 and Sox17 expression were unaffected (Supplementary Figure 18).

In addition, we examined brain sections from the parietal lobe of 24 schizophrenia donor brains and 24 age-matched normal control brains (Supplementary Figure 19). We stained for claudin-5 levels in brain sections, and assessed pattern and density of staining in a blinded manner. While overall levels of protein were not changed, an aberrant pattern of claudin-5 immunoreactivity was observed in 62% of the schizophrenia cases—usually claudin-5 is continuously expressed across the length of a blood vessel, but was discontinuous in many of the samples taken from individuals with schizophrenia (Figure 5f). Quantification of claudin-5 expression in blood vessels revealed that, while there were no significant differences between the diagnosis groups in the absence of the rs10314 variant, claudin-5 levels were significantly lower in individuals with a diagnosis of schizophrenia who had the rs10314 variant compared to control subjects who harboured the rs10314 variant (*P<0.05, Figure 5g).

## Discussion

Current treatment options for schizophrenia patients largely and almost exclusively include the use of anti-psychotic therapies and adjunct psychosocial therapy, including psychotherapy and cognitive behavioural therapy. However, anti-psychotic drugs are often discontinued by patients due to inefficacy or intolerable side effects. In addition, the average life expectancy of people with schizophrenia can be between 10–25 years less than normal, with a recent meta-analysis suggesting up to 16 000 people die annually as a result of having to live with the condition.^35^ There is now a clear and urgent need to better understand the underlying molecular aetiology of schizophrenia and to develop new forms of therapy for this debilitating condition.

To our knowledge, this is the first molecular-based evidence of the involvement of the BBB to be described in schizophrenia, and brings together an environmental- and genetic-based model for the molecular aetiology of schizophrenia. The protein product of the *claudin-5* gene is a four pass transmembrane protein consisting of two extracellular loops that reside at the apical periphery of contacting endothelial cells of the BBB. These extracellular loops can respond to the microenvironment and can allow for rapid remodelling of the TJ dependent on a range of environmental stimuli.^36^

The underlying molecular complexity of the BBB and how it relates to health and disease is only beginning to be unravelled. Indeed, the BBB is not a static microenvironment, it is highly dynamic in both homoeostatic physiology and indeed in pathology. At the BBB, claudin-5 is by far the dominant TJ component, but claudin-3 and claudin-12 are also present. Our understanding of the claudins has been considerably improved through genetic knockout models. In particular, the role of claudin-5 in forming the BBB was confirmed in mice genetically engineered to lack claudin-5. Nitta *et al.* ^33^ showed that claudin-5 knockout mice have an impaired BBB that was compromised in a size-selective manner to molecules up to 800 Da in size. However, complete ablation of claudin-5 is lethal, with mice dying within hours of birth. Intriguingly, however, in claudin-5 knockout mice, a barrier can still form and remains intact to molecules >~1 kDa molecular weight. Our data obtained from inducible ‘knockdown’ mice now suggests a link between the gene-dosage effect of claudin-5 and the onset of schizophrenia-like characteristics in this mouse model. This does not by any means suggest that variations in claudin-5 is a direct cause of schizophrenia, but that it may be a contributing factor in the development of schizophrenia. It is known that claudin-5 levels are impacted in other neurological disorders such as ischaemia and traumatic brain injury^17^ and neurodegenerative disorders.^37,38,39^

While the original studies describing the association of the rs10314 variant in the 3′-UTR of claudin-5 with schizophrenia in the general population were weak, our studies here showing a nominal association in a cohort of individuals with 22q11DS with a single remaining claudin-5 allele are important. The central role of claudin-5 at the BBB cannot be underestimated and it has been identified as an ohnologue, or a gene duplicate originating from whole-genome duplication. Pertinently, ohnologues represent critical dosage-sensitive elements of the genome, responsible for some of the deleterious phenotypes observed for pathogenic copy-number variations and as such are readily identifiable candidate genes for schizophrenia.^40,41^

Critically, it appears that some of the most common anti-psychotic drugs can potently regulate claudin-5 protein levels in a dose-dependent manner. These findings are important given the fact that brain endothelial cells are the first membranous interface these drugs encounter in the cerebrum post administration. This is the first example of these drugs being biologically active in brain endothelial cells and may be fundamental to their mode of action. This is highly suggestive that these drugs can regulate the integrity of the BBB and may go some way to explaining why select drugs have efficacy in some patients and not others. In addition, this suggests that claudin-5 may be a therapeutic target for schizophrenia, with drugs that regulate its expression representing a more targeted and ultimately safer approach to treatment.

At the anatomical level, the central pathological findings in the brains of schizophrenia patients are centred on a distinct loss of cortical grey matter, cortical thinning and reduced numbers of synaptic structures on cortical pyramidal neurons.^42^ There are however no pathognomonic markers of schizophrenia observable at the histological level post-mortem. Here, we were able to identify an aberrant pattern of claudin-5 immunoreactivity in 62% of schizophrenia patient brains based on an analysis of claudin-5 levels.

Recognizing that schizophrenia is a disorder with a cerebral vascular component will impact the way this condition is treated and lead to improved medicines for patients living with the condition. These findings will also lead to a greater understanding of other neuropsychiatric conditions such as bipolar disorder and affective disorders.

## Electronic supplementary material

Supplementary Information

Supplementary Figure 1

Supplementary Figure 2

Supplementary Figure 3

Supplementary Figure 4

Supplementary Figure 5

Supplementary Figure 6

Supplementary Figure 7

Supplementary Figure 8

Supplementary Figure 9

Supplementary Figure 10

Supplementary Figure 11

Supplementary Figure 12

Supplementary Figure 13

Supplementary Figure 14

Supplementary Figure 15

Supplementary Figure 16

Supplementary Figure 17

Supplementary Figure 18

Supplementary Figure 19

Supplementary Table 1

Supplementary Table 2

Supplementary video 1

Supplementary video 2

Supplementary Movie Legends

## Electronic supplementary material

**Supplementary Information** accompanies the paper on the *Molecular Psychiatry* website 10.1038/mp.2017.156

## References

[1] World Health Organization, World Health Organization, Apr. 2016, retrieved from: www.who.int/mediacentre/factsheets/fs397/en/. Schizophrenia.

[2] Lewis DA, Lieberman JA (2000). Catching up on schizophrenia: natural history and neurobiology. Neuron.

[3] Palmer BA, Pankratz VS, Bostwick JM (2005). The lifetime risk of suicide in schizophrenia: a re-examination. Arch Gen Psychiatry.

[4] McGlashan TH (1996). Early detection and intervention in schizophrenia research. Schizophr Bull.

[5] Voruganti L, Cortese L, Oyewumi L, Cernovsky Z, Awad A (2000). Comparative evaluation of conventional and novel antipsychotic drugs with reference to their subjective tolerability, side-effect profile and impact on quality of life. Schizophr Res.

[6] Leucht S, Corves C, Arbter D, Engel RR, Li C, Davis JM (2009). Second-generation versus first-generation antipsychotic drugs for schizophrenia: a meta-analysis. Lancet.

[7] Pulver AE (2000). Search for schizophrenia vulnerability genes. Biol Psychiatry.

[8] Liu H, Heath SC, Sobin C, Roos JL, Galke BL, Blundell ML (2002). Genetic variation at the 22q11 PRODH2/DGCR6 locus presents an unusual pattern and increases susceptibility to schizophrenia. Proc Natl Acad Sci USA.

[9] Rees E, Kirov G, Sanders A, Walters JT, Chambert KD, Shi J (2014). Evidence that duplications of 22q11.2 protect against schizophrenia. Mol Psychiatry.

[10] Murphy KC (2002). Schizophrenia and velo-cardio-facial syndrome. Lancet.

[11] Williams NM (2011). Molecular mechanisms in 22q11 deletion syndrome. Schizophr Bull.

[12] Kao A, Mariani J, McDonald-McGinn DM, Maisenbacher MK, Brooks-Kayal AR, Zackai EH (2004). Increased prevalence of unprovoked seizures in patients with a 22q11.2 deletion. Am J Med Genet A.

[13] Tang SX, Yi JJ, Calkins ME, Whinna DA, Kohler CG, Souders MC (2013). Psychiatric disorders in 22q11.2 deletion syndrome are prevalent but undertreated. Psychol Med.

[14] Abbott NJ, Patabendige AA, Dolman DE, Yusof SR, Begley DJ (2010). Structure and function of the blood-brain barrier. Neurobiol Dis.

[15] Abbott NJ, Ronnback L, Hansson E (2006). Astrocyte-endothelial interactions at the blood-brain barrier. Nat Rev Neurosci.

[16] Pardridge WM (2005). The blood-brain barrier: bottleneck in brain drug development. NeuroRx.

[17] Hawkins BT, Davis TP (2005). The blood-brain barrier/neurovascular unit in health and disease. Pharmacol Rev.

[18] Obermeier B, Daneman R, Ransohoff RM (2013). Development, maintenance and disruption of the blood-brain barrier. Nat Med.

[19] Falcone T, Fazio V, Lee C, Simon B, Franco K, Marchi N (2010). Serum S100B: a potential biomarker for suicidality in adolescents?. PLoS ONE.

[20] Falcone T, Carlton E, Lee C, Janigro M, Fazio V, Forcen FE (2015). Does systemic inflammation play a role in pediatric psychosis?. Clin Schizophr Relat Psychoses.

[21] Keaney J, Walsh DM, O'Malley T, Hudson N, Crosbie DE, Loftus T (2015). Autoregulated paracellular clearance of amyloid-β across the blood-brain barrier. Sci Adv.

[22] Keaney J, Campbell M (2015). The dynamic blood-brain barrier. FEBS J.

[23] Lui J, Castelli LM, Pizzinga M, Simpson CE, Hoyle NP, Bailey KL (2014). Granules harboring translationally active mRNAs provide a platform for P-body formation following stress. Cell Rep.

[24] Nagy A, Gertsenstein M, Vintersten K, Behringer R. *Manipulating the Mouse Embryo: A Laboratory Manual*. Cold Spring Harbor, Laboratory Press, 2003.

[25] Dickins RA, McJunkin K, Hernando E, Premsrirut PK, Krizhanovsky V, Burgess DJ (2007). Tissue-specific and reversible RNA interference in transgenic mice. Nat Genet.

[26] Seibler J, Kleinridders A, Kuter-Luks B, Niehaves S, Bruning JC, Schwenk F (2007). Reversible gene knockdown in mice using a tight, inducible shRNA expression system. Nucleic Acids Res.

[27] Premsrirut PK, Dow LE, Kim SY, Camiolo M, Malone CD, Miethingi C (2011). A rapid and scalable system for studying gene function in mice using conditional RNA interference. Cell.

[28] Sun ZY, Wei J, Xie L, Shen Y, Liu SZ, Ju GZ (2004). The CLDN5 locus may be involved in the vulnerability to schizophrenia. Eur Psychiatry.

[29] Ye L, Sun Z, Xie L, Liu S, Ju G, Shi J (2005). Further study of a genetic association between the CLDN5 locus and schizophrenia. Schizophr Res.

[30] Wu N, Zhang X, Jin S, Liu S, Ju G, Wang Z (2010). A weak association of the CLDN5 locus with schizophrenia in Chinese case-control samples. Psychiatry Res.

[31] Omidinia E, Mashayekhi Mazar F, Shahamati P, Kianmehr A, Shahbaz Mohammadi H (2014). Polymorphism of the CLDN5 gene and schizophrenia in an Iranian population. Iran J Public Health.

[32] Campbell M, Humphries MM, Kiang AS, Nguyen AT, Gobbo OL, Tam LC (2011). Systemic low-molecular weight drug delivery to pre-selected neuronal regions. EMBO Mol Med.

[33] Nitta T, Hata M, Gotoh S, Seo Y, Sasaki H, Hashimoto N (2003). Size-selective loosening of the blood-brain barrier in claudin-5-deficient mice. J Cell Biol.

[34] Harris LW, Wayland M, Lan M, Ryan M, Giger T, Lockstone H (2008). The cerebral microvasculature in schizophrenia: a laser capture microdissection study. PLoS ONE.

[35] Lieberman JA, Stroup TS, McEvoy JP, Swartz MS, Rosenheck RA, Perkins DO (2005). Effectiveness of antipsychotic drugs in patients with chronic schizophrenia. N Engl J Med.

[36] Haseloff RF, Dithmer S, Winkler L, Wolburg H, Blasig IE (2015). Transmembrane proteins of the tight junctions at the blood-brain barrier: structural and functional aspects. Semin Cell Dev Biol.

[37] Mandel I, Paperna T, Glass-Marmor L, Volkowich A, Badarny S, Schwartz I (2012). Tight junction proteins expression and modulation in immune cells and multiple sclerosis. J Cell Mol Med.

[38] Garbuzova-Davis S, Sanberg PR (2014). Blood-CNS barrier impairment in ALS patients versus an animal model. Front Cell Neurosci.

[39] Doherty CP, O'Keefe E, Wallace E, Loftus T, Keaney J, Kealy J (2016). Blood-brain barrier dysfunction as a hallmark pathology in chronic traumatic encephalopathy. J Neuropathol Exp Neurol.

[40] McLysaght A, Makino T, Grayton HM, Tropeano M, Mitchell KJ, Vassos E (2014). Ohnologs are overrepresented in pathogenic copy number mutations. Proc Natl Acad Sci USA.

[41] Takashi M, McLysaght A (2010). Ohnologs in the human genome are dosage balanced and frequently associated with disease. Proc Natl Acad Sci USA.

[42] Sekar A, Bialas AR, de Rivera H, Davis A, Hammond TR, Kamitaki N (2016). Schizophrenia risk from complex variation of complement component 4. Nature.

