# Supplementary material for: Dose-dependent expression of claudin-5 is a modifying factor in schizophrenia
Source: Mol Psychiatry. 2017 Oct 10;23(11):2156–66. doi: 10.1038/mp.2017.156 (PMC6298981; doi:10.1038/mp.2017.156)
Supplement: Supplementary file 1 — Supplementary Information [file 41380_2018_149_MOESM1_ESM.doc]

**Supplementary Figure Legends**

**Supplementary Figure 1:** Derived allele frequencies for 262 individuals from 113 populations taken from the Simons Genome Diversity Project (SGDP). Populations are represented by circles, with allele frequencies indicated through colour intensity. The number of individuals in each population is represented by circle size, with the majority of populations containing just two individuals.

**Supplementary Figure 2:** Frequencies in 2504 individuals from the 1000 Genomes Project. Populations are grouped and coloured with reference to the 1000 Genomes continental super population to which they belong. Derived allele frequencies are represented through pie-charts, labelled according to 1000 Genomes population codes. Super population derived allele frequencies are also noted.

**Supplementary Figure 3:** **A)** Claudin-5 expression in Caco-2 cells expressing normal or rs10314 variant claudin-5. **B)** Levels of claudin-5 transcript remain un-changed.

**Supplementary Figure 4: a)** GFP and isolectin IB4 staining in the hippocampus of a mouse injected with a doxycycline-inducible GFP expressing AAV. **b)** Graph showing the percentage of isolectin IB4+/GFP+vessels. **c)** Suppression of claudin-5 did not lead to angiogenesis – quantification of total blood vessel length showed no significant differences between control and claudin-5-suppressed groups. **d)** Examples of leakage of biotinylated agent at low magnification in the mPFC.

**Supplementary Figure 5:** Suppression of claudin-5 had no significant effect on occludin or ZO-1 levels in the **A)** mPFC or **B)** hippocampus.

**Supplementary Figure 6:** Experiments investigating the effect of hippocampal claudin-5 suppression on learning and memory. **a)** There were no significant differences between treatment groups in the Y-maze in a spontaneous alternation task. However, claudin-5 suppression was associated with a non-significant trend (P = 0.0633) towards a side bias in the task which may reflect subtle changes in hippocampal function. **b)** There were no significant differences between treatment groups in the alternating T-maze. However, claudin-5 suppression was associated with a non-significant trend (P = 0.0637) towards reduced choice times in the task, again perhaps reflecting subtle changes in hippocampal function. **c)** There were no significant differences between the treatment groups in the object recognition task. **d)** There were no significant differences between treatment groups in the radial arm maze. **e)** There were no significant differences between treatment groups in a retention probe trial in the radial arm maze (+10 days following the end of the standard radial arm maze protocol).

**Supplementary Figure 7:** Experiments investigating the effect of hippocampal claudin-5 suppression on affect and social behaviour. **a)** There were no significant differences between treatment groups in the splash test. **b)** There was a significant impairment following claudin-5 suppression in the hippocampus in the social novelty task (*P = 0.0148). **c)** There was a significant delay in the latency to begin grooming in the open field test (*P = 0.0226) and a non-significant trend towards decreased overall grooming time (P = 0.0780) in the open field test. There were no significant effects on time spent in the centre zone or in the amount of time spent freezing. **d)** There were no significant differences between treatment groups in the forced swim test. **e)** There were no significant differences between treatment groups in the elevated plus maze.

**Supplementary Figure 8:** Experiments investigating the effect of hippocampal claudin-5 suppression on motor co-ordination and locomotor behaviour. **a)** There were no significant differences between treatment groups on locomotor activity in the open field test. However, there was a non-significant trend towards increased rearing (P = 0.0735) following claudin-5 suppression. **b)** There were no significant differences between treatment groups on the Rota-Rod. **c)** There were no significant differences between treatment groups in average speed during the radial arm maze.

**Supplementary Figure 9:** Experiments investigating the effect of claudin-5 suppression in the mPFC on learning and memory. **a)** There were no significant differences between treatment groups in the Y-maze in a spontaneous alternation task. **b)** There was a significant impairment in performance in the alternating T-maze following claudin-5 suppression (*P = 0.0173). **c)** There was a significant impairment in performance in the object recognition task following claudin-5 suppression (**P = 0.0019). **d)** There were no significant differences between treatment groups in the radial arm maze. **e)** There were no significant differences between treatment groups in a retention probe trial in the radial arm maze (+10 days following the end of the standard radial arm maze protocol).

**Supplementary Figure 10:** Experiments investigating the effect of claudin-5 suppression in the mPFC on affect and social behaviour. **a)** There were no significant differences between treatment groups in the splash test. **b)** There were no significant differences between treatment groups in either the social preference or social novelty tasks. **c)** There were no significant differences between treatment groups in grooming, time in the centre zone or time spent freezing in the open field test. **d)** There was a significant reduction in time spent immobile in the forced swim test following claudin-5 suppression (***P < 0.0003). **e)** There were no significant differences between treatment groups in the elevated plus maze.

**Supplementary Figure 11:** Experiments investigating the effect of claudin-5 suppression in the mPFC on motor co-ordination and locomotor behaviour. **a)** There were no significant differences between treatment groups on locomotor activity in the open field test. **b)** There were no significant differences between treatment groups on the Rota-Rod. **c)** There were no siginificant differences between treatment groups in average speed during the radial arm maze.

**Supplementary Figure 12:** Experiments investigating the effect of claudin-5 suppression on learning and memory in an inducible claudin-5 knockdown mouse strain. **a)** There was a significant side bias observed following claudin-5 suppression in a spontaneous alternation task in the Y-maze (*P = 0.0120; see Fig. 4a). However, this had no significant effect on overall performance in the task. **b)** There were no significant differences between groups on trial completion times in the alternating T-maze following claudin-5 suppression.

**Supplementary Figure 13:** Experiments investigating the effect of claudin-5 suppression on affect and social behaviour in an inducible claudin-5 knockdown mouse strain. **a)** There were no significant differences between groups in the splash test. **b)** There were no significant 3differences between groups in either the social preference or social novelty tasks. **c)** There were no significant differences between groups in grooming, time in the centre zone or time spent freezing in the open field test. **d)** There were no significant differences between groups in the forced swim test.

**Supplementary Figure 14:** Experiments investigating the effect of claudin-5 suppression on motor co-ordination and locomotor behaviour in an inducible claudin-5 knockdown mouse strain. **a)** There were no significant differences between treatment groups on locomotor activity in the open field test. However, there were non-significant trends towards an increase in distance travelled over time (P = 0.0567) and an increase in average speed following claudin-5 suppression (P = 0.0674). **b)** There were no significant differences between treatment groups on the Rota-Rod.

**Supplementary Figure 15:** Electroretinography (ERG) tracings in the right eye (top tracing) and left eye (bottom tracing) in 5 inducible claudin-5 knockdown mice.

**Supplementary Figure 16:** There were significantly increased levels of fibrinogen extravasation in the **a)** cortex (**P < 0.01) and **b)** hippocampus in the inducible claudin-5 knockdown mouse.

**Supplementary Figure 17:** Antipsychotic treatment also affected the expression of other tight junction proteins. **a-c)** Lithium and haloperidol significantly increased levels of occludin *in vitro* (*P < 0.05). **d-f)** Chlorpromazine significantly increased levels of ZO-1 *in vivo* (*P < 0.05).

**Supplementary Figure 18:** Expression of Axin-2 and Sox17 24 h following antipsychotic treatment in mice.

**Supplementary Figure 19:** **a)** Parietal lobe of donor human brain. **b)** Details of normal and schizophrenia human brain tissues used in this study.

**Supplementary Methods**

*Extraction of free and formulated siRNA at 100 µg/mL in AGM plasma*

Free claudin-5 siRNA and PEI formulated siRNA were spiked into ice-cold or 37 ^o^C African Green Monkey plasma (K2EDTA), respectively, to obtain samples containing Claudin-5 siRNA at final concentration of 100 µg/mL. The samples were extracted in six replicates. Samples were subsequently analyzed by HPLC/MS.

*Behavioural Experiments:*

*1. General information and test schedule:*

Mice were all handled for approximately 5 minutes per day for 1 week prior to behavioural testing. Except where stated, mice had free access to food and water throughout the testing schedule. Before each test, mice were taken from their holding room and allowed to habituate to the testing room for 5-10 minutes prior to testing. All behavioural experiments were performed during the light phase and all apparatus were cleaned with 70 % ethanol before use and between trials, except where stated. Statistical analysis was performed using Prism 5 for Mac OS X (GraphPad Software, Inc., U.S.A.) and *p* < 0.05 was taken as being statistically significant in all cases.

The test schedule for mice that were stereotaxically injected with an AAV was as follows:

The test schedule for the inducible mice was as follows:

*2. Object recognition task:*

Long-term recognition memory was assessed using the object recognition task (1). The object recognition task was performed in a plastic rectangular arena (38 x 43 x 18 cm). Three similarly sized objects were used for all mice (plastic culture tube filled with NaCl; brown glass bottle filled with water; 50 ml plastic tube filled with solution of bromophenol blue) with animals showing no preference for one particular object over the others. The task consisted of two 3-minute sessions:

1. Familiarisation session: Two objects are positioned at two fixed locations within the testing arena.
2. Test session: One of the objects from the Familiarisation session is placed in the same location as before (Familiar object) and the second object is replaced with a third object (Novel object).

At the beginning of each session, the test mouse was placed in the arena facing the wall and positioned equidistant from the two objects. The timer was started when the experimenter released the mouse’s tail and the number of nose contacts with each object was counted. Between sessions, the arena and objects were cleaned with 70 % ethanol and the mouse was returned to its home cage for the intersession interval (ISI) of 3 hours. Each mouse’s performance was assessed by calculating their discrimination index during the test session:

with Nose contacts_Novel_ being the number of nose contacts with the novel object and Nose contacts_Familiar_ being the number of nose contacts with the familiar object from the familiarisation session. Positive values for the Discrimination index are associated with a preference for the novel object (the expected behaviour) and negative values are associated with a preference for the familiar object. Values that tend close to 0 mean that the mouse has no preference for one object over the other. Each experimental group was compared to its relevant control group via an unpaired *t*-test.

*3. Splash Test:*

Induced grooming behaviour was assessed using the splash test (2). Individual mice were left in their home cage for the duration of the splash test (5 minutes); cage mates were temporarily stored in a separate cage. The mouse was sprayed on its dorsal side with a 10 % sucrose solution and a timer was started. At the end of the test, the mouse was removed to the temporary holding cage and the next mouse was placed in the home cage. Video recordings were made of the entire trial, including the spraying procedure. The videos were then analysed by an experimenter who was blind to the experimental condition looking at two measures:

1. Latency to begin grooming: The time (seconds) taken for the mouse to initiate any form of grooming behaviour.
2. Total grooming time: The total time (seconds) that the mouse engaged in any form of grooming behaviour.

Each experimental group was compared to its relevant control group via an unpaired *t*-test.

*4. RotaRod:*

Motor co-ordination was assessed using the RotaRod (3). Mice were run on the RotaRod (Ugo Basile, Italy) along with all their cage mates to reduce anxiety in performing the task. The RotaRod was started at a constant speed of 4 rotations per minute (rpm) and each mouse from the cage was placed on a lane in the RotaRod. Once all the mice were in position, the RotaRod was set to accelerate from 4 to 60 rpm over 3 minutes. Two measures were taken:

1. The time (seconds) at which the mouse fell from its lane.
2. The acceleration (rpm) at the time at which the mouse fell from its lane.

Each experimental group was compared to its relevant control group via an unpaired *t*-test. The RotaRod was cleaned with 70 % ethanol between each trial and reset to 4 rpm for the next cage.

*5. Y-Maze:*

Working spatial memory was assessed using the spontaneous alternation on a Y-Maze (4). Individual mice were placed in the centre zone of a Perspex Y-Maze (three 30 x 5 cm joined by a 20 cm diameter central area; bounded by wall 15 cm high) and allowed to freely explore the apparatus for 8 minutes. Arm entries were counted by the experimenter when the mouse placed all four paws into an arm, delineated by the slot for guillotine doors associated with the apparatus (the doors themselves were not used in this task). Each arm entry was recorded in the order in which they occurred. Spontaneous alternation was assessed after the experiment was over; a successful alternation being defined as the mouse entering all three arms of the Y-Maze over any 4-arm entry span (e.g. if the mouse entered the arms in the order 1, 2, 1, 3, 1, 3 then it would count as two alternations out of a possible three). Errors were counted if the mouse exited an arm and then returned to that arm before visiting either of the other two arms. Each experimental group was compared to its relevant control group via an unpaired *t*-test.

*6. Elevated Plus Maze:*

Anxiety-like behaviour was assessed using the elevated plus maze (5). Individual mice were placed in the centre zone of a Perspex elevated plus maze (four 30 x 5 cm arms; two open arms and two closed arms with wall height = 20 cm; apparatus raised on Perspex legs to a height of 40 cm) and allowed to freely explore the apparatus for 10 minutes. Arm entries were logged by a computerized tracking system (ANY-Maze, Version 4.99m, Stoelting Co., U.S.A.) on a Hewlett-Packard ProBook running Windows 8.1 with the total closed arm entries and open arm entries being taken for analysis. Levels of anxiety-like behaviour were analyzed by comparing the number of entries into the open arms between the groups. Each experimental group was compared to its relevant control group via an unpaired *t*-test.

*7. Forced Swim Test:*

Depression-like behaviour was assessed using the forced swim test (6). A large clear plastic beaker (diameter 17 cm; height 21 cm) was filled with lukewarm water (20-21 °C). Mice were slowly placed individually into the water and a timer was started at the moment where the experimenter released the mouse’s tail. Mice were allowed to swim for 6 minutes and were then removed from the apparatus and dried off with paper towel. Mice were then allowed to dry off under a heating lamp before returned to the cage rack. Video recordings were made covering the placement of the mouse into the apparatus up until the end of the swimming period. The videos were then analyzed by an experimenter who was blind to the experimental condition, recording the total time (in seconds) spent engaged in escape behaviour during the final 4 minutes of the swimming period (6). This value was subtracted from the total time (240 seconds) to give the time spent immobile. Each experimental group was compared to its relevant control group via an unpaired *t*-test.

*8. T-Maze:*

Working spatial memory was assessed using the alternating T-Maze paradigm (7). Mice were individually placed into the start arm of a Perspex T-Maze (three arms 30 x 10 cm; wall height 20 cm) with a guillotine door preventing them from accessing the maze. Each mouse underwent 10 sessions with each session comprising of two trials. One session was performed in the morning and another in the evening over 5 days. During the first trial in each session, a Perspex divider was present to encourage the selection of one arm over the other. The trial began when the guillotine door in the start arm was removed and a stopwatch was started at the same time. The stopwatch was stopped when the mouse entered one of the choice arms. An arm entry was defined as the point when entire mouse, including its tail, was in the arm and at this point a guillotine door was placed at the entrance of the arm to prevent the mouse from going back into the start arm. The time taken to enter an arm and the name of the arm selected (A or B) was noted by the experimenter and the guillotine door to the start arm was replaced. The mouse was then returned to the start arm for the second trial.

Before beginning the second trial, the divider between the two choice arms and the guillotine arm from the choice arm from the first trial was removed. The apparatus was not cleaned between trials, as mice should use olfactory cues to make a choice on this task (see Ref. 7). The second trial again began when the guillotine door for the start arm was removed and ended when the mouse fully entered one of the two arms, again using a stopwatch to time performance in the task. The mouse was contained in the choice arm with a guillotine door before being returned to its home cage. The time taken to enter the arm and the name of the arm selected was again noted. Each mouse was scored for each session using a binary system: 1 for alternating between the arms; and 0 for visiting the same arm twice in a session. The time taken to enter an arm on the second trial of each session was also analyzed as a response time. Each experimental group was compared to its relevant control group via unpaired *t*-tests.

*9. Open Field Test:*

General locomotor activity and anxiety-like behaviour was assessed using the open field test (8). Mice were individually placed into a Perspex open field arena (30 x 30 x 21 cm) and allowed to freely explore for 30 minutes. The mice were tracked using a computerized tracking system (ANY-Maze, Version 4.99m, Stoelting Co., U.S.A.) on a Hewlett-Packard ProBook running Windows 8.1, which automatically recorded distance, average speed, time spent in the three zones (outer perimeter; middle perimeter; centre zone) and time spent freezing. The experimenter overseeing the test used hot keys to record the time spent rearing and grooming during the 30 minutes test. Each experimental group was compared to its relevant control group via unpaired *t*-tests for measures made over the total time and via repeated measures analyses of variance (ANOVAs) for data from the three zones and for data that had been time-binned into 5 minute intervals.

*10. Social Behaviour Tests:*

Social behaviour was assessed using the social preference and social novelty tasks (9). The apparatus used was a plastic arena (38 x 43 x 18 cm) divided into three areas by Perspex dividers. The central start zone was empty but the two zones on either side contained circular dome-shaped transparent plastic housing containers (diameter 15 cm; height 9 cm) that had a number of air holes drilled around their circumference. There were three trials in every session and each mouse was given one session each. In the habituation trial, mice were placed individually into the central start zone and allowed to freely explore the empty apparatus for 5 minutes. The mouse being tested was then removed and placed in a temporary holding container while the experimenter wiped down the apparatus with 70 % ethanol and then placed an unfamiliar mouse from another holding room into one of the two housing containers. For the social preference trial, the mouse being tested was placed in the start zone and allowed to explore the apparatus (containing one empty housing container and one with an unfamiliar mouse in it) for 10 minutes. Again, the mouse being tested was placed in a temporary holding container while the experimenter wiped down the apparatus with 70 % ethanol. Another unfamiliar mouse was then placed the previously empty housing container. For the social novelty trial, the mouse being tested was placed in the start zone and allowed to explore the apparatus (now containing two housing containers; one containing a familiar mouse from the social preference trial and the other containing a novel mouse) for 10 minutes. At the end of the session, all mice were returned to their respective home cages and all of the apparatus was cleaned with 70 % ethanol before setting up for the next mouse to be tested. In the social preference and social novelty trials, the mice were tracked using a computerised tracking system (ANY-Maze, Version 4.99m, Stoelting Co., U.S.A.) on a Hewlett-Packard ProBook running Windows 8.1. The time (in seconds) spent in the area around each housing container was measured and a social interaction index was calculated for each trial. For the social preference trial, the social interaction index was calculated as:

with Time_Mouse_ being the time spent in the zone surrounding the unfamiliar mouse and Time_Empty_ being the time spent in the zone surrounding the empty housing container. For the social novelty trial, the social interaction index was calculated as:

*12. Radial Arm Maze:*

Working and long-term spatial memory was assessed using the 8-armed radial arm maze (10). In order to familiarize the mice with the food rewards that were used the radial arm maze (chocolate flavoured cereal, Aldi, Ireland), the food rewards were introduced to their home cages on two occasions in the week preceding the radial arm maze in order to minimize any neophobic responses to the food rewards in the maze. In order to encourage the mice to search for the food rewards, they were placed on a restricted diet (approximately 80-85 % of free-feeding weight) for three days prior to starting the radial arm maze. The radial arm maze consisted of two phases:

1. Standard radial arm maze protocol of one trial per day over 10 days.
2. A single retention trial 10 days after the final trial of the standard protocol.

The restricted diet was kept in place for the duration of the standard radial arm maze protocol with *ad libitum* access to food reintroduced following the final trial. The restricted diet was started again three days prior to the retention trial and *ad libitum* access to food reintroduced following the retention trial. On the day before starting the standard radial arm maze protocol, mice were habituated to the Perspex radial arm maze (eight 30 x 5 cm joined by a 20 cm diameter central area; bounded by wall 15 cm high); mice were placed on the maze with their cage mates and allowed to freely explore the apparatus in groups for 5 minutes. This was to minimize any anxiety effects during learning in the radial arm maze.

On the test days, the radial arm maze was baited with four food rewards. These were each placed into four of the eight arms, leaving four empty arms. The food rewards were put into recessed food wells so that mice could not see whether a food reward was present or not until they had traversed down the arm to directly check the well. Each mouse had its own pattern for baiting with the four baited arms being pseudorandomly assigned before the experiment started and the same arms were baited for each mouse across all trials. The radial arm maze was cleaned with 70 % ethanol between all trials and after placement of the food rewards to minimize olfactory cues.

For each trial, mice were individually placed in the central start zone of the radial arm maze and allowed to explore until they had consumed all four of the food rewards or until the trial had run for 15 minutes. At the end of the trial, mice were returned to their home cage. All trials were recorded using a computerised tracking system (ANY-Maze, Version 4.99m, Stoelting Co., U.S.A.) on a Hewlett-Packard ProBook running Windows 8.1 with the number of arm entries, average speed and trial duration being measured. Working memory errors were counted when a mouse entered an arm that it had already visited during that trial and reference memory errors (reflecting long-term storage of food reward locations) were counted when a mouse entered an arm that does not contain a food reward. Each experimental group was compared to its relevant control group via unpaired *t*-tests for measures made over the total experiment and via repeated measures analyses of variance (ANOVAs) for data that had been analysed on a trial-by-trial basis. The first two trials were considered as training trials and were not included in the statistical analysis of the task (10).

For the retention trial, mice were run in exactly the same manner in order to see whether treatment affected retention of the food reward locations over several days of rest. Again, working and reference memory errors were analysed along with average speed and trial duration. Each experimental group was compared to its relevant control group via an unpaired *t*-test.

**Allelic frequencies of rs10314 in diverse modern populations**

Two separate modern datasets were used to explore derived allele frequencies at rs10314 in modern populations. The first comprised of 2504 individuals belonging to 26 populations from the 1000 Genomes Phase 3 cohort [(1000 Genomes Project Consortium et al. 2015)](https://paperpile.com/c/aHkxwI/wNsk)). Allele frequencies for these populations were calculated based on genotypes reported by 1000 Genomes. The second was based on a combination of two recently published genome diversity panels alongside nine individuals published in [(Raghavan et al. 2015)](https://paperpile.com/c/aHkxwI/sl2j). Genotypes for 402 modern individuals from the Estonian Biocentre human Genome Diversity Panel (EGDP), published in [(Pagani et al. 2016)](https://paperpile.com/c/aHkxwI/9P6q) were downloaded from <http://evolbio.ut.ee/CGgenomes.html>. Genotypes for a further 288 modern individuals from the Simon’s Genome Diversity Project (SGDP) [(Mallick et al. 2016; Prüfer et al. 2014)](https://paperpile.com/c/aHkxwI/OhHD+FE6z) were downloaded from <https://www.simonsfoundation.org/life-sciences/simons-genome-diversity-project/> and <http://reichdata.hms.harvard.edu/pub/datasets/sgdp/>, resulting in a final dataset of 699 genotypes. Individuals were divided into large groupings based on geographical region. These groups were then further segregated based on sub-region. Finally, where possible, populations were defined within these sub-regions based on language grouping. Derived allele frequencies for populations, sub-regions and regions are shown in Table 1.

**Supplementary References**

1. Leger M, Quiedeville A, Bouet V, Haelewyn B, Boulouard M, Schumann-Bard P, Freret T. Object recognition test in mice. Nat Protoc. 2013 Dec;8(12):2531-7.
2. Yalcin I, Aksu F, Belzung C. Effects of desipramine and tramadol in a chronic mild stress model in mice are altered by yohimbine but not by pindolol. Eur J Pharmacol. 2005 May 9;514(2-3):165-74.
3. Deacon RM. Measuring motor coordination in mice. J Vis Exp. 2013 May 29;(75):e2609.
4. Maurice T, Hiramatsu M, Itoh J, Kameyama T, Hasegawa T, Nabeshima T. Behavioral evidence for a modulating role of sigma ligands in memory processes. I. Attenuation of dizocilpine (MK-801)-induced amnesia. Brain Res. 1994 May 30;647(1):44-56.
5. Komada M, Takao K, Miyakawa T. Elevated plus maze for mice. J Vis Exp. 2008 Dec 22;(22). pii: 1088.
6. Can A, Dao DT, Arad M, Terrillion CE, Piantadosi SC, Gould TD. The mouse forced swim test. J Vis Exp. 2012 Jan 29;(59):e3638.
7. Deacon RM, Rawlins JN. T-maze alternation in the rodent. Nat Protoc. 2006;1(1):7-12.
8. Bailey KR, Crawley JN. Anxiety-Related Behaviors in Mice. In: *Methods of Behavior Analysis in Neuroscience*, (2nd edition), Buccafusco JJ (Ed.). Boca Raton (FL): CRC Press/Taylor & Francis; 2009.
9. Kaidanovich-Beilin O, Lipina T, Vukobradovic I, Roder J, Woodgett JR. Assessment of social interaction behaviors. J Vis Exp. 2011 Feb 25;(48). pii: 2473.
10. Crusio WE, Schwegler H, Brust I. Covariations between hippocampal mossy fibres and working and reference memory in spatial and non-spatial radial maze tasks in mice. Eur J Neurosci. 1993 Oct 1;5(10):1413-20.
11. [Hofmanová, Zuzana, Susanne Kreutzer, Garrett Hellenthal, Christian Sell, Yoan Diekmann, David Díez-Del-Molino, Lucy van Dorp, et al. 2016. “Early Farmers from across Europe Directly Descended from Neolithic Aegeans.” *Proceedings of the National Academy of Sciences of the United States of America* 113 (25): 6886–91.](http://paperpile.com/b/aHkxwI/xDR7)
12. [Günther, Torsten, Cristina Valdiosera, Helena Malmström, Irene Ureña, Ricardo Rodriguez-Varela, Óddny Osk Sverrisdóttir, Evangelia A. Daskalaki, et al. 2015. “Ancient Genomes Link Early Farmers from Atapuerca in Spain to Modern-Day Basques.” *Proceedings of the National Academy of Sciences*, September. doi:](http://paperpile.com/b/aHkxwI/XIXj)[10.1073/pnas.1509851112](http://dx.doi.org/10.1073/pnas.1509851112)[.](http://paperpile.com/b/aHkxwI/XIXj)
13. [Li, Heng, and Richard Durbin. 2009. “Fast and Accurate Short Read Alignment with Burrows–Wheeler Transform.” *Bioinformatics*  25 (14): 1754–60.](http://paperpile.com/b/aHkxwI/AOjg)
14. [Li, Heng, Bob Handsaker, Alec Wysoker, Tim Fennell, Jue Ruan, Nils Homer, Gabor Marth, Goncalo Abecasis, Richard Durbin, and 1000 Genome Project Data Processing Subgroup. 2009. “The Sequence Alignment/Map Format and SAMtools.” *Bioinformatics*  25 (16): 2078–79.](http://paperpile.com/b/aHkxwI/Ijua)
15. [1000 Genomes Project Consortium, Adam Auton, Lisa D. Brooks, Richard M. Durbin, Erik P. Garrison, Hyun Min Kang, Jan O. Korbel, et al. 2015. “A Global Reference for Human Genetic Variation.” *Nature* 526 (7571): 68–74.](http://paperpile.com/b/aHkxwI/wNsk)
16. [Browning, Sharon R., and Brian L. Browning. 2007. “Rapid and Accurate Haplotype Phasing and Missing-Data Inference for Whole-Genome Association Studies by Use of Localized Haplotype Clustering.” *American Journal of Human Genetics* 81 (5): 1084–97.](http://paperpile.com/b/aHkxwI/wJ8t)
