# Supplementary figures and images for: Dose-dependent expression of claudin-5 is a modifying factor in schizophrenia
Source: Mol Psychiatry. 2017 Oct 10;23(11):2156–66. doi: 10.1038/mp.2017.156 (PMC6298981; doi:10.1038/mp.2017.156)

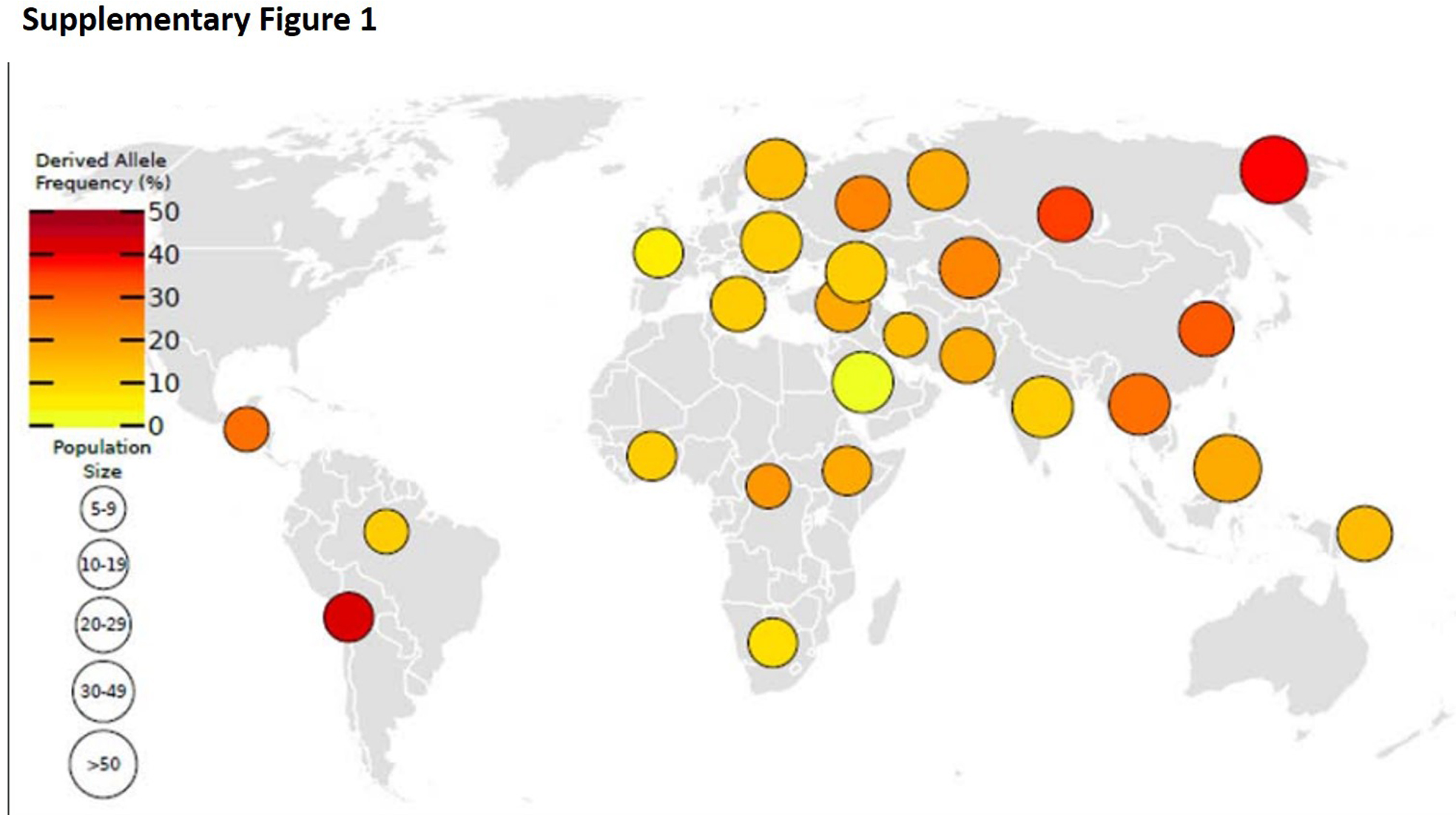

Supplement: Supplementary file 2 — Supplementary Figure 1 [file 41380_2018_149_MOESM2_ESM.jpg]

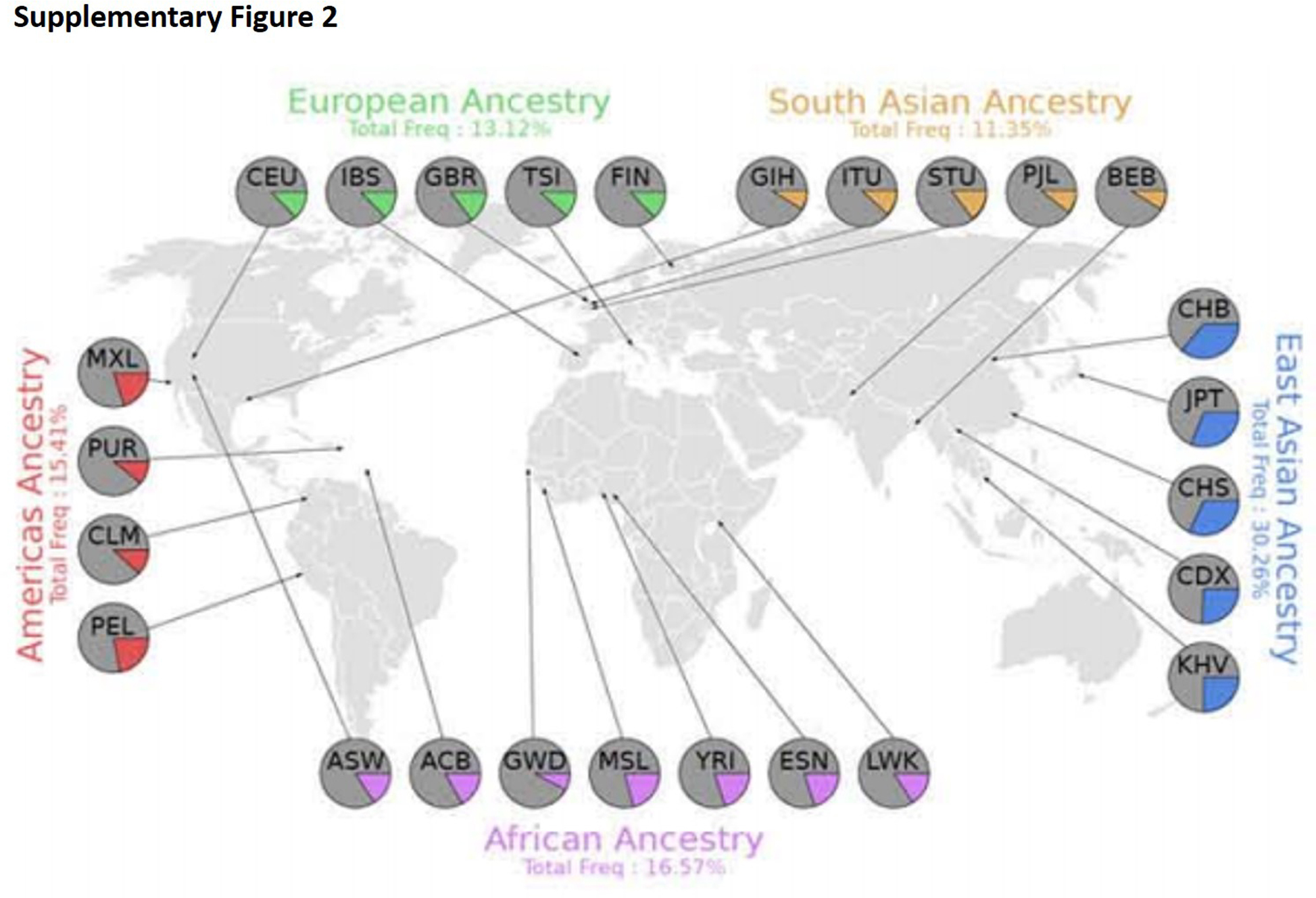

Supplement: Supplementary file 3 — Supplementary Figure 2 [file 41380_2018_149_MOESM3_ESM.jpg]

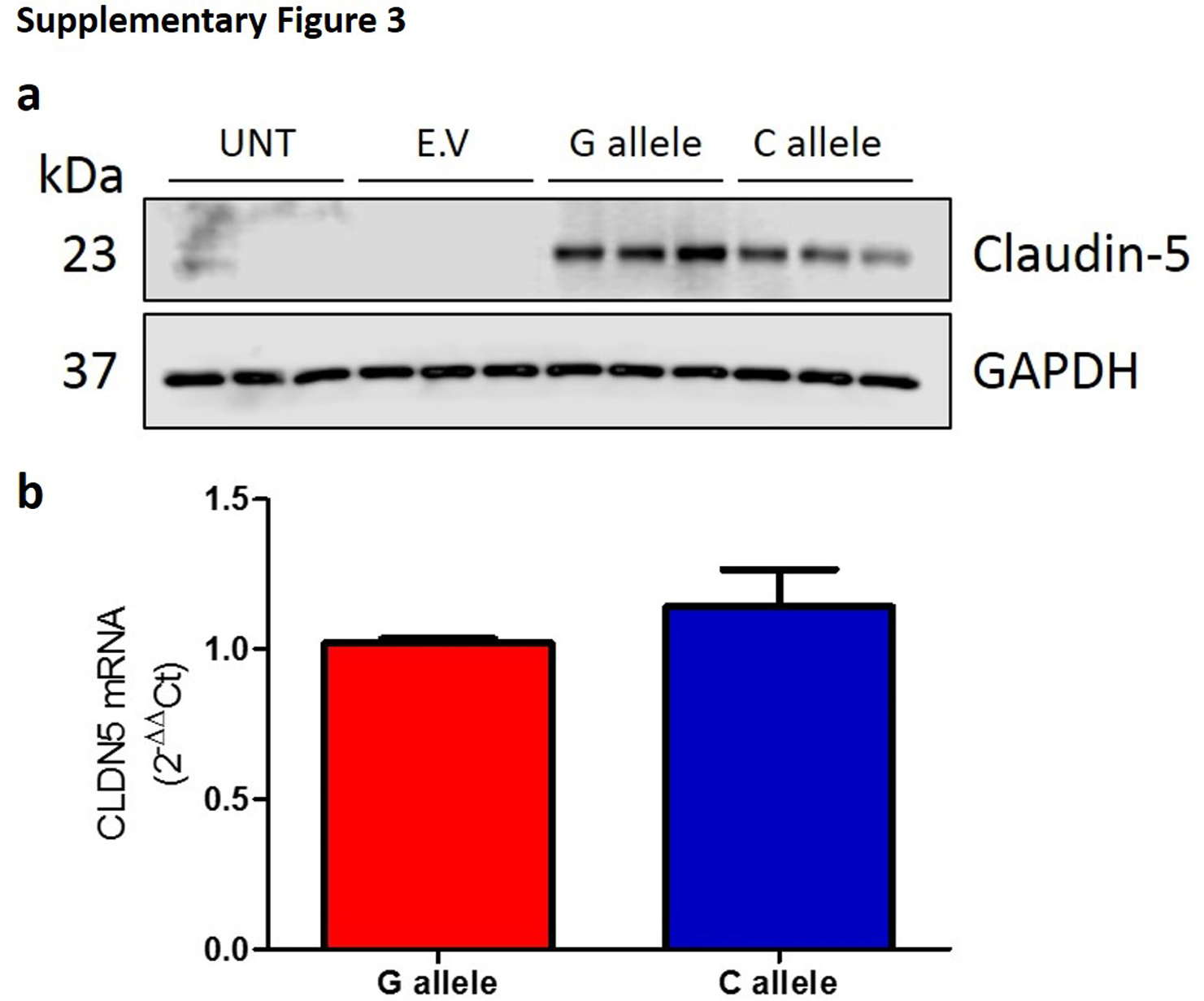

Supplement: Supplementary file 4 — Supplementary Figure 3 [file 41380_2018_149_MOESM4_ESM.jpg]

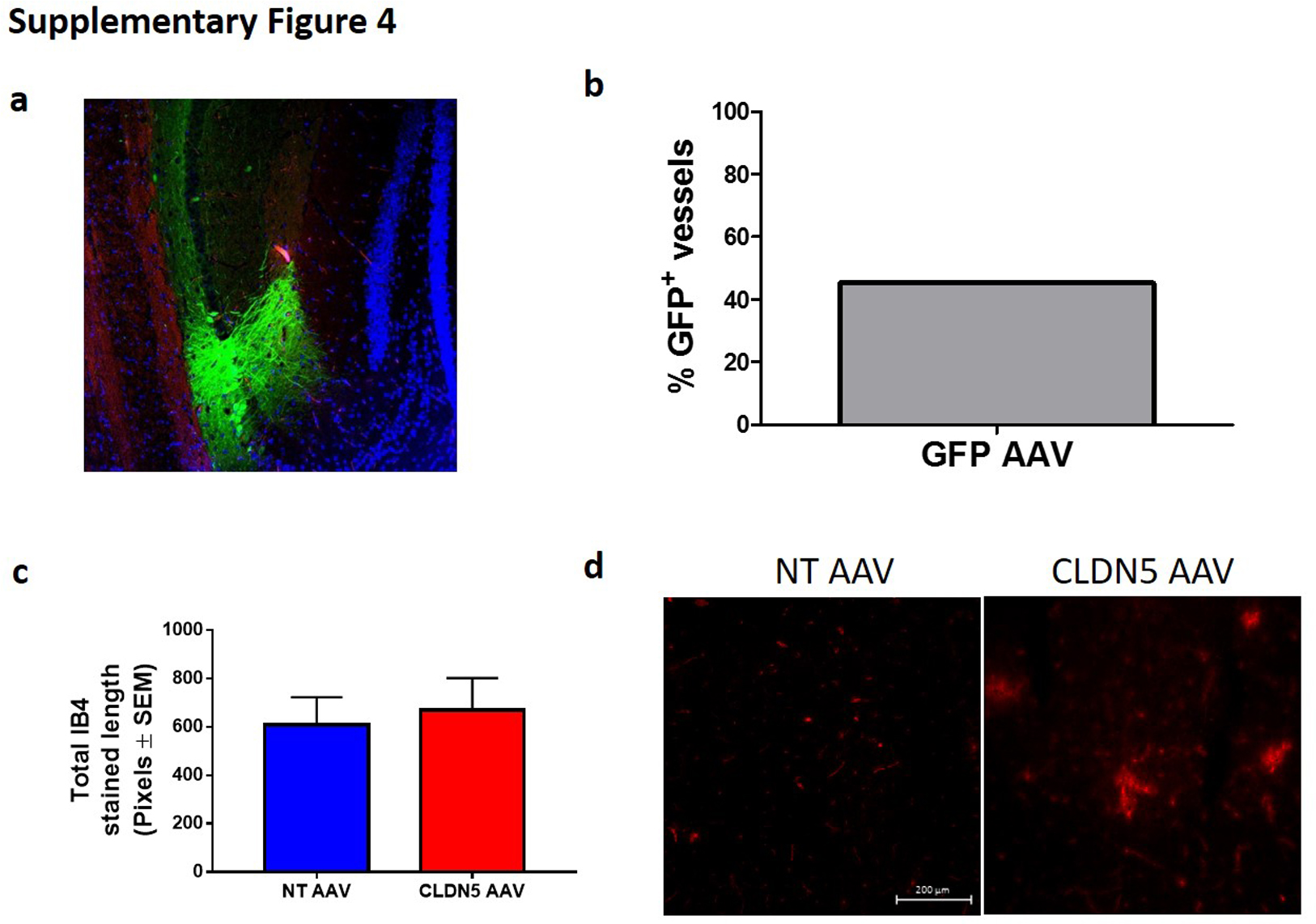

Supplement: Supplementary file 5 — Supplementary Figure 4 [file 41380_2018_149_MOESM5_ESM.jpg]

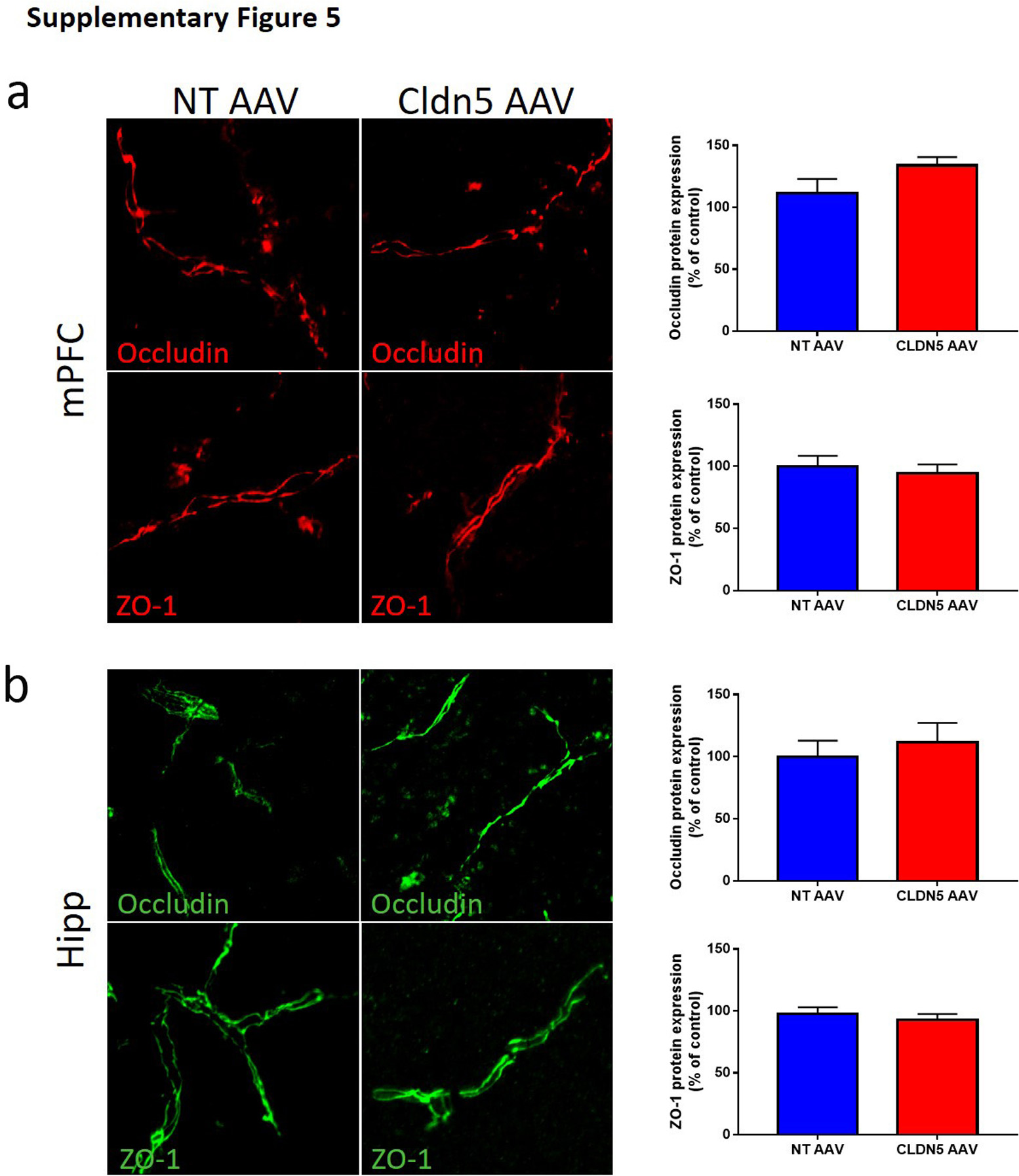

Supplement: Supplementary file 6 — Supplementary Figure 5 [file 41380_2018_149_MOESM6_ESM.jpg]

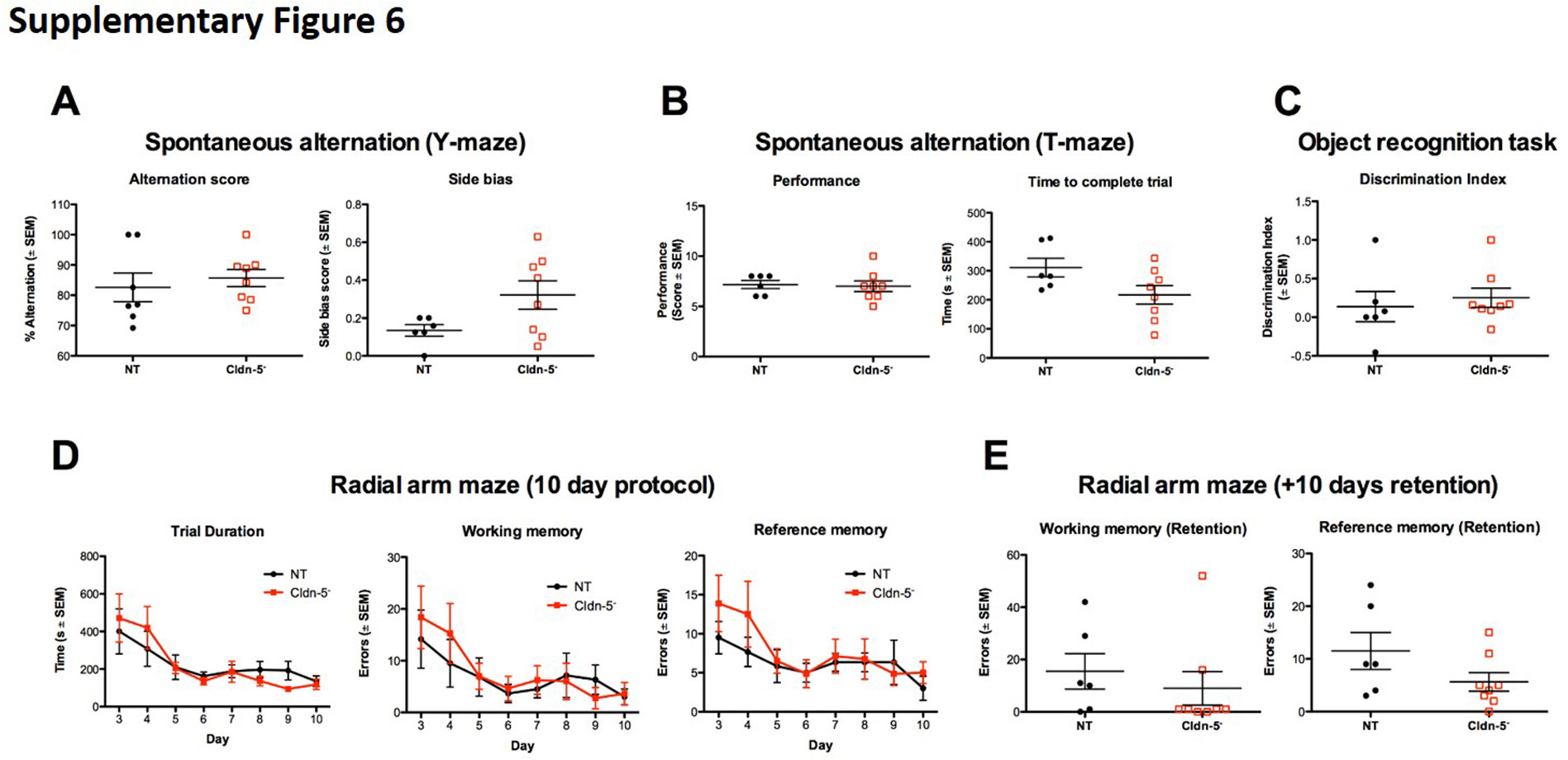

Supplement: Supplementary file 7 — Supplementary Figure 6 [file 41380_2018_149_MOESM7_ESM.jpg]

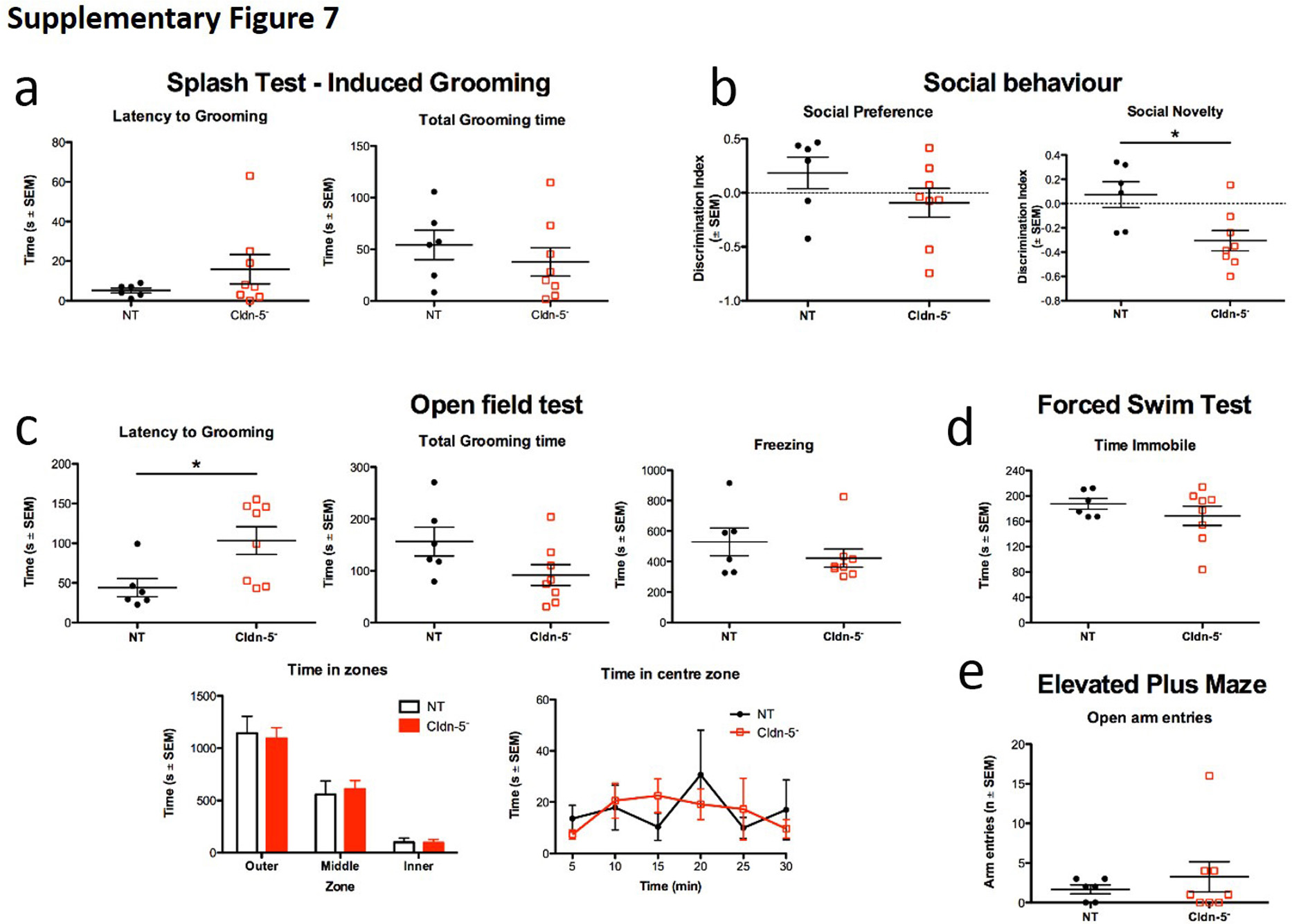

Supplement: Supplementary file 8 — Supplementary Figure 7 [file 41380_2018_149_MOESM8_ESM.jpg]

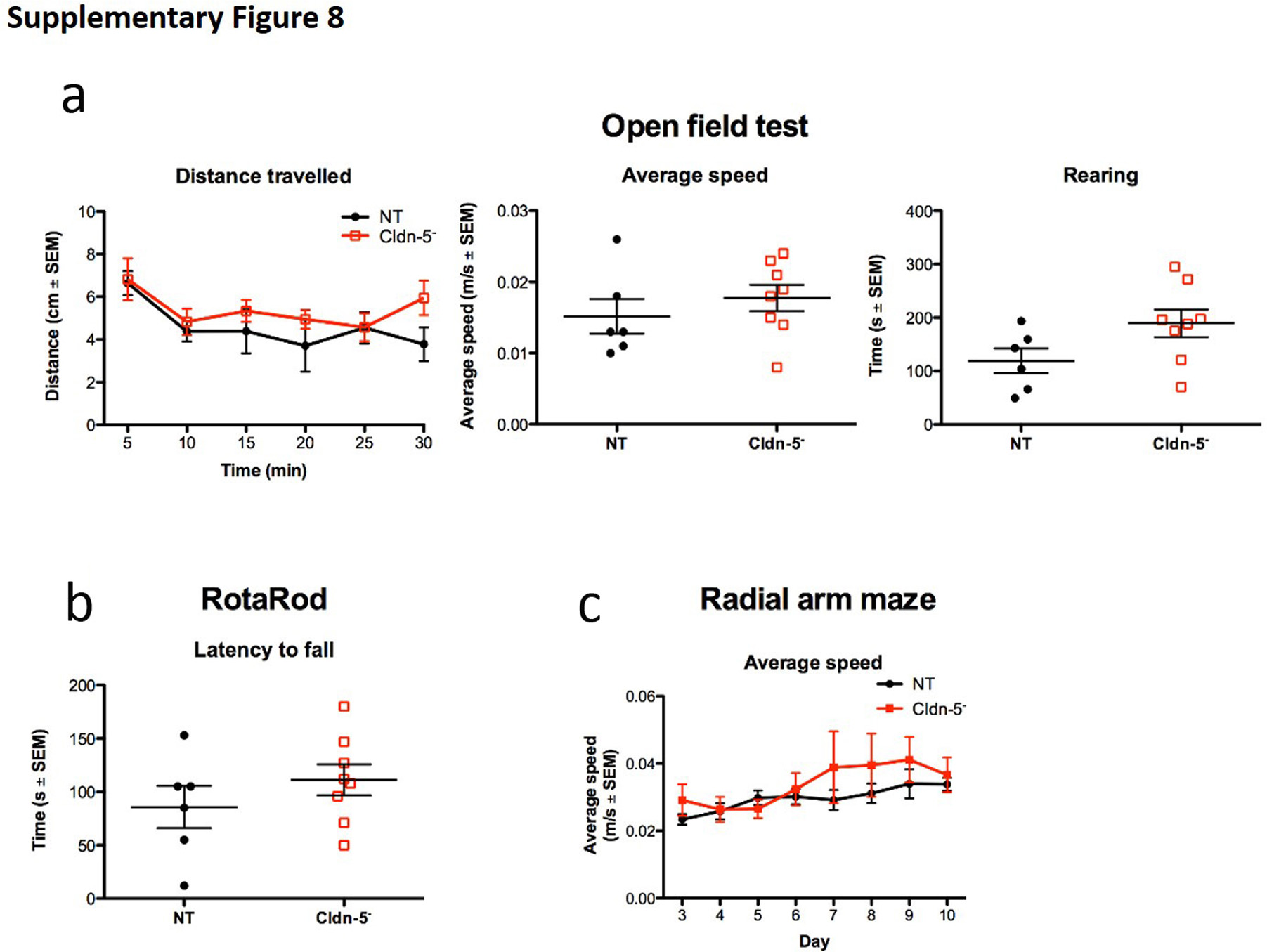

Supplement: Supplementary file 9 — Supplementary Figure 8 [file 41380_2018_149_MOESM9_ESM.jpg]

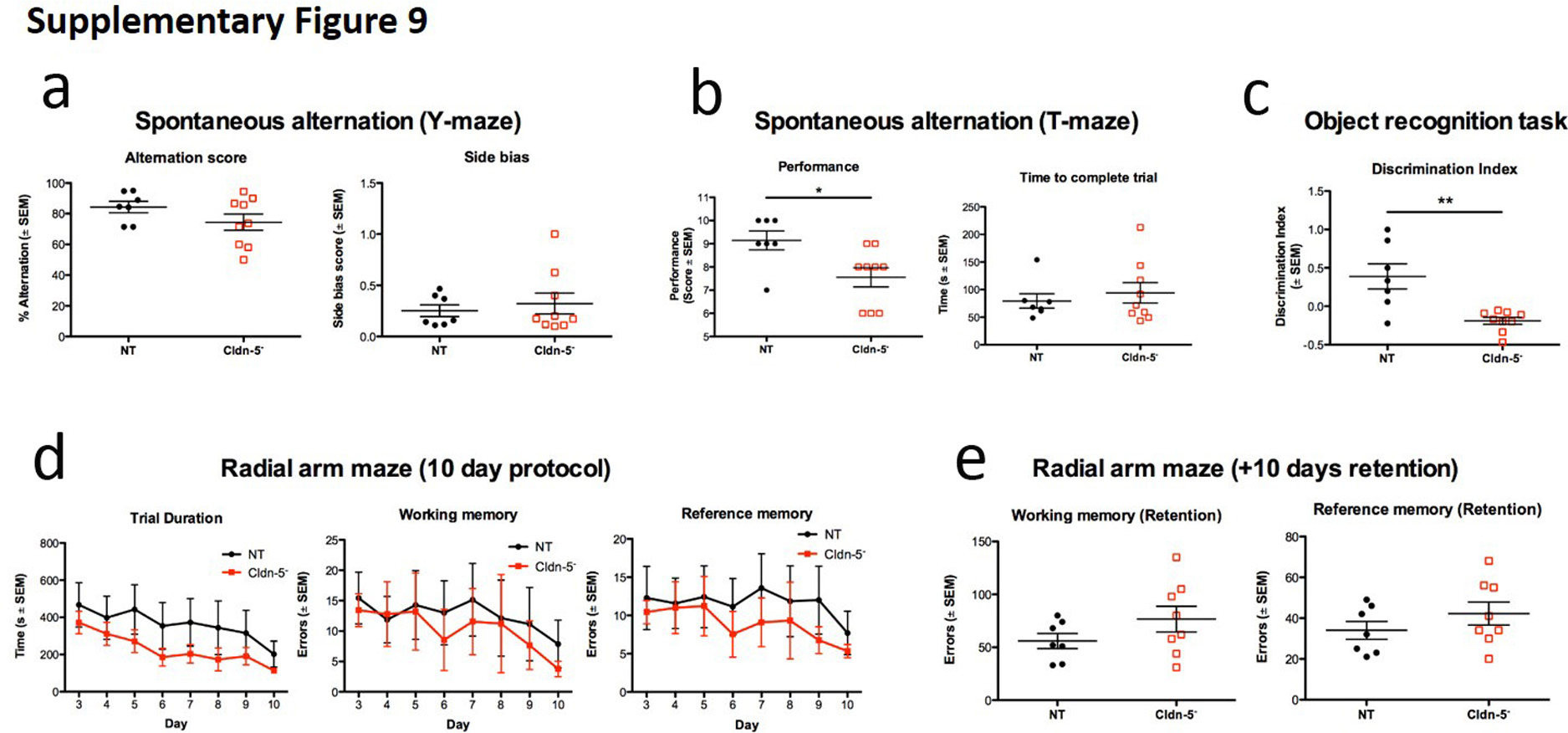

Supplement: Supplementary file 10 — Supplementary Figure 9 [file 41380_2018_149_MOESM10_ESM.jpg]

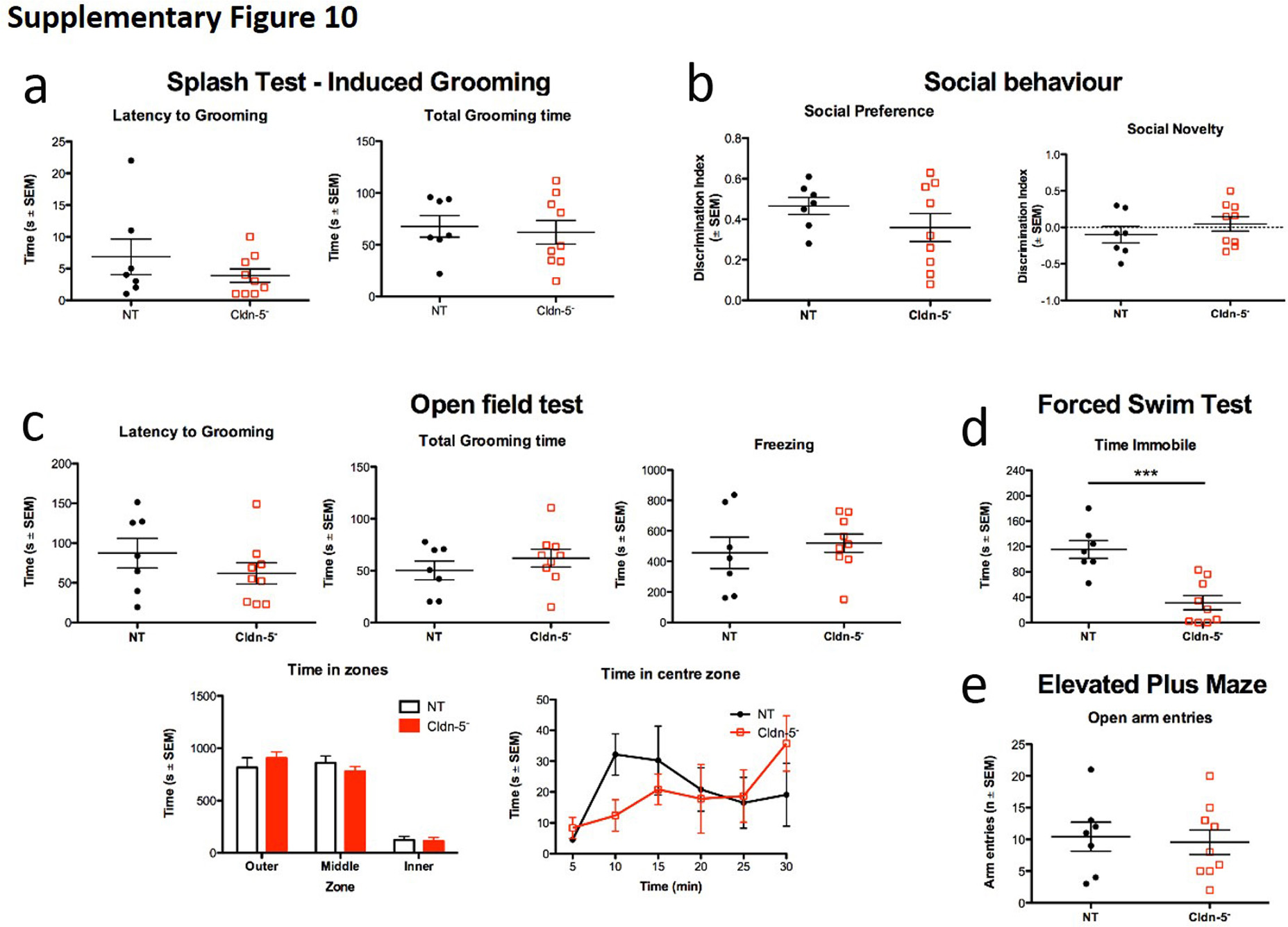

Supplement: Supplementary file 11 — Supplementary Figure 10 [file 41380_2018_149_MOESM11_ESM.jpg]

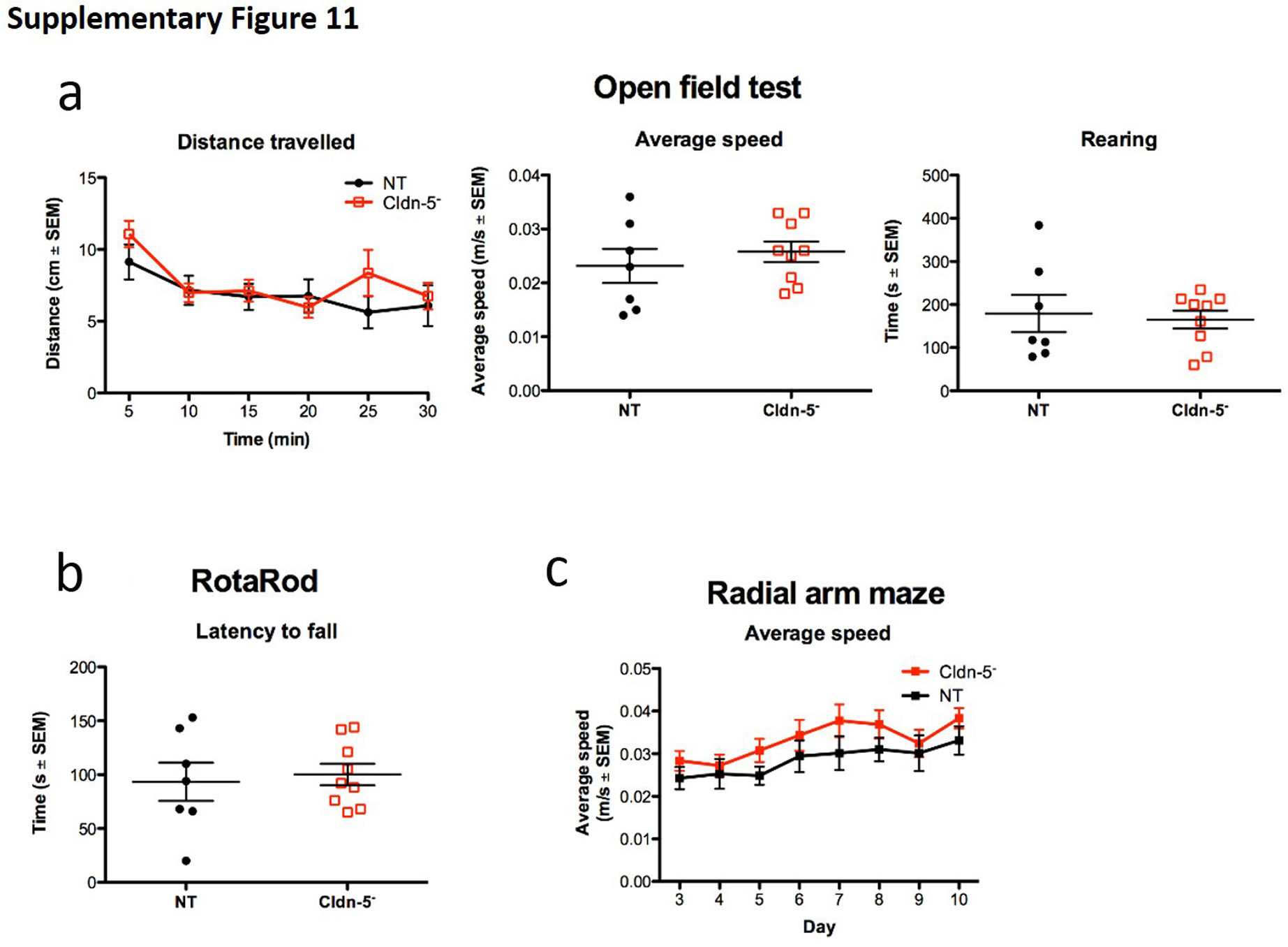

Supplement: Supplementary file 12 — Supplementary Figure 11 [file 41380_2018_149_MOESM12_ESM.jpg]

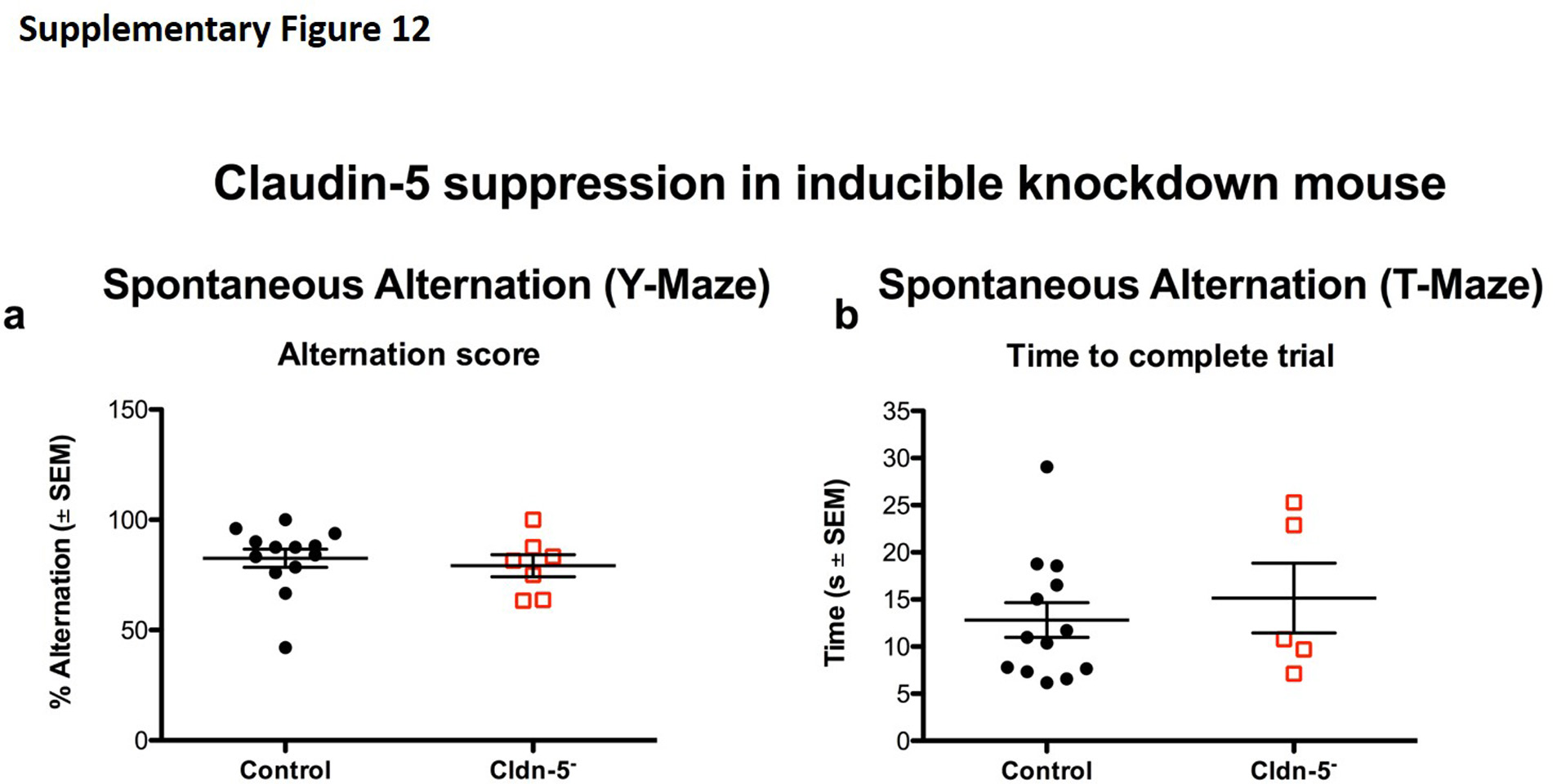

Supplement: Supplementary file 13 — Supplementary Figure 12 [file 41380_2018_149_MOESM13_ESM.jpg]

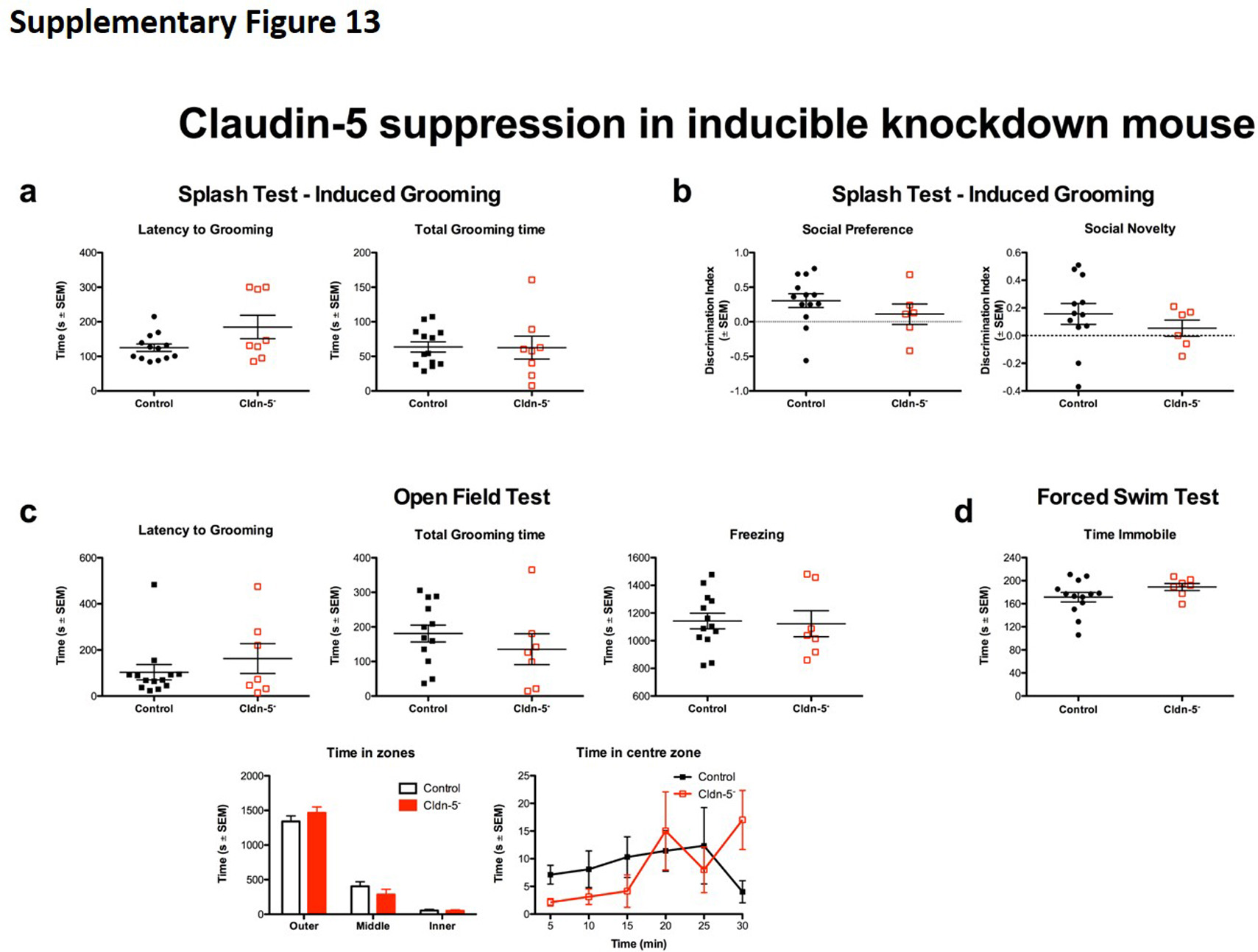

Supplement: Supplementary file 14 — Supplementary Figure 13 [file 41380_2018_149_MOESM14_ESM.jpg]

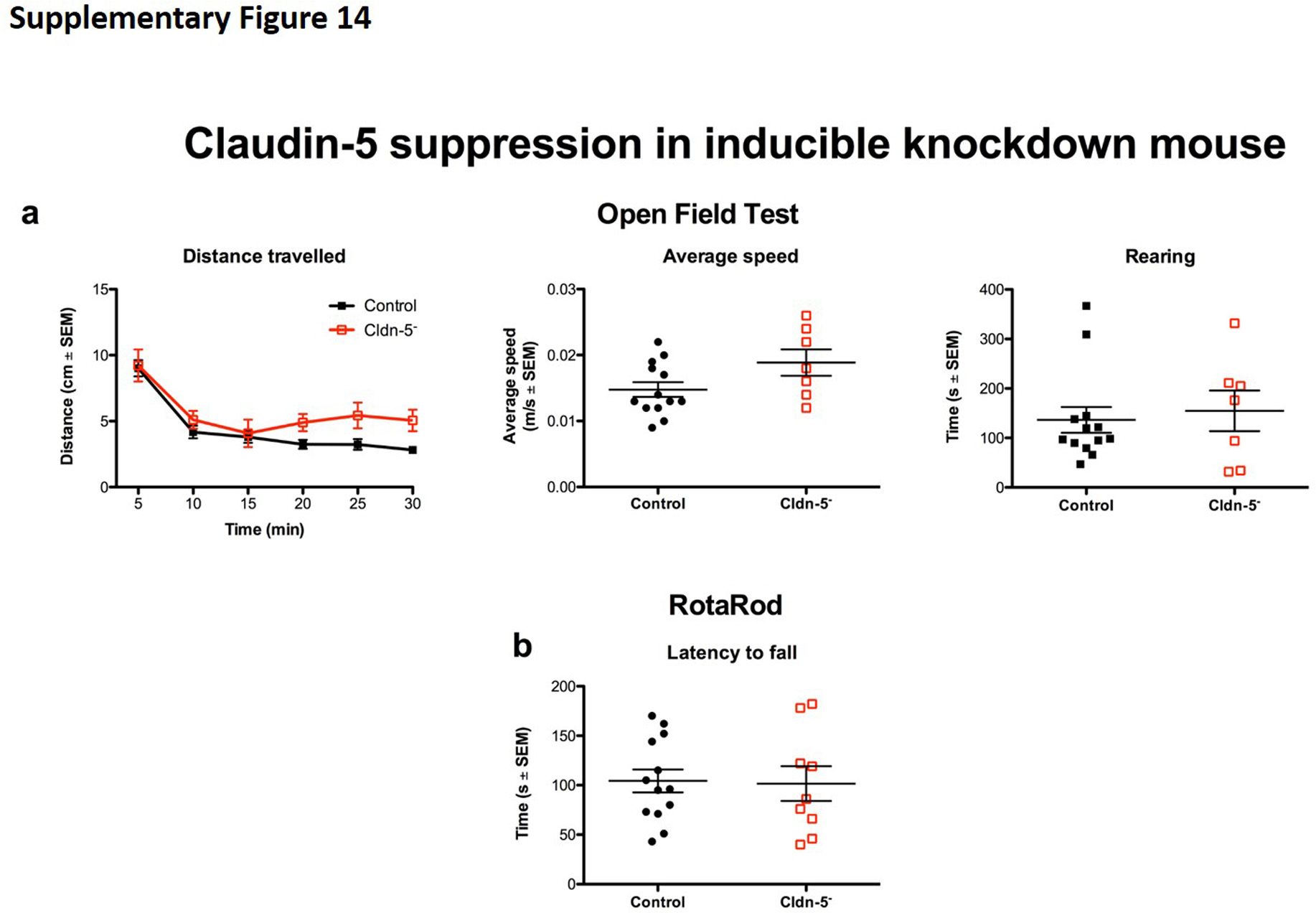

Supplement: Supplementary file 15 — Supplementary Figure 14 [file 41380_2018_149_MOESM15_ESM.jpg]

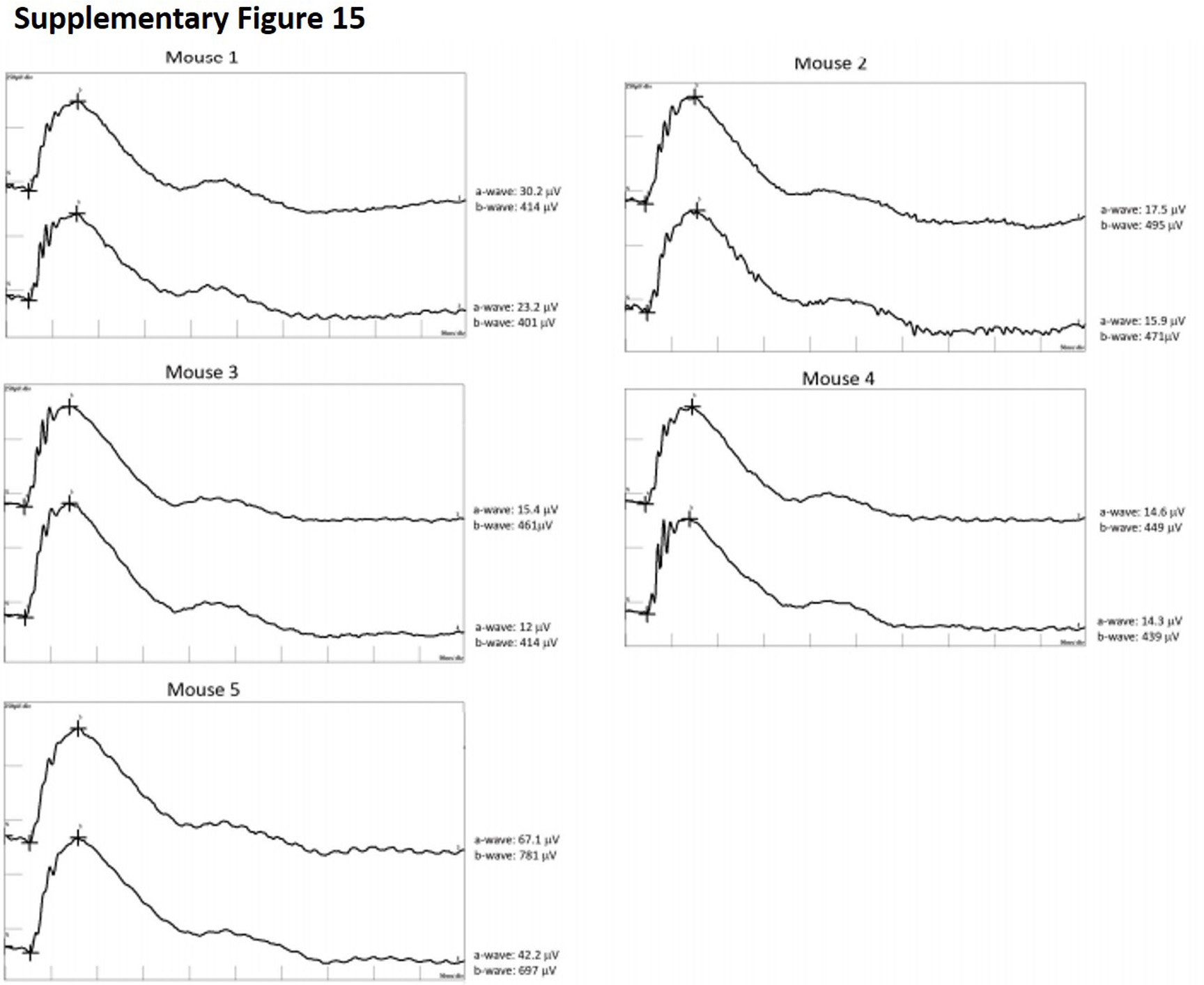

Supplement: Supplementary file 16 — Supplementary Figure 15 [file 41380_2018_149_MOESM16_ESM.jpg]

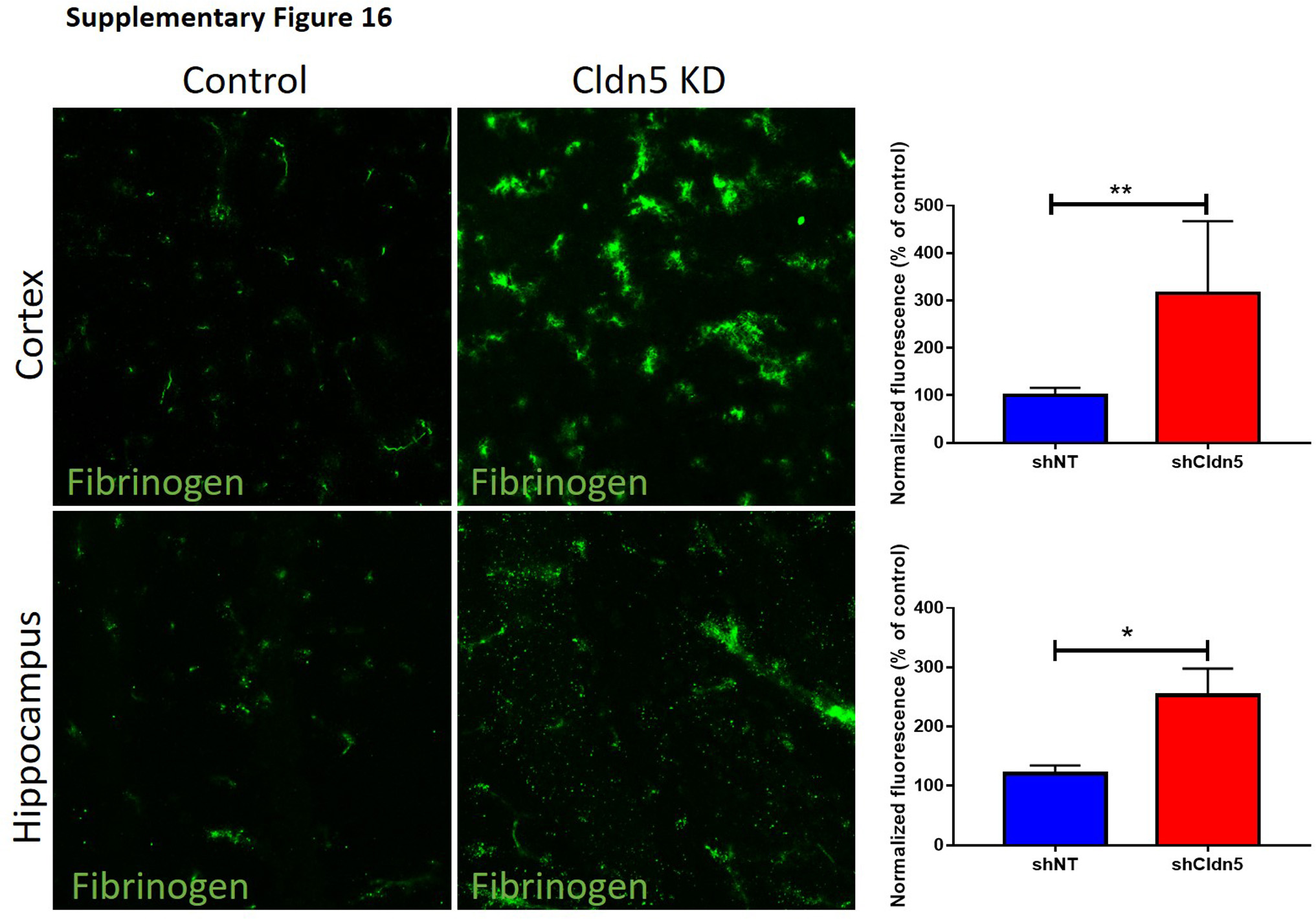

Supplement: Supplementary file 17 — Supplementary Figure 16 [file 41380_2018_149_MOESM17_ESM.jpg]

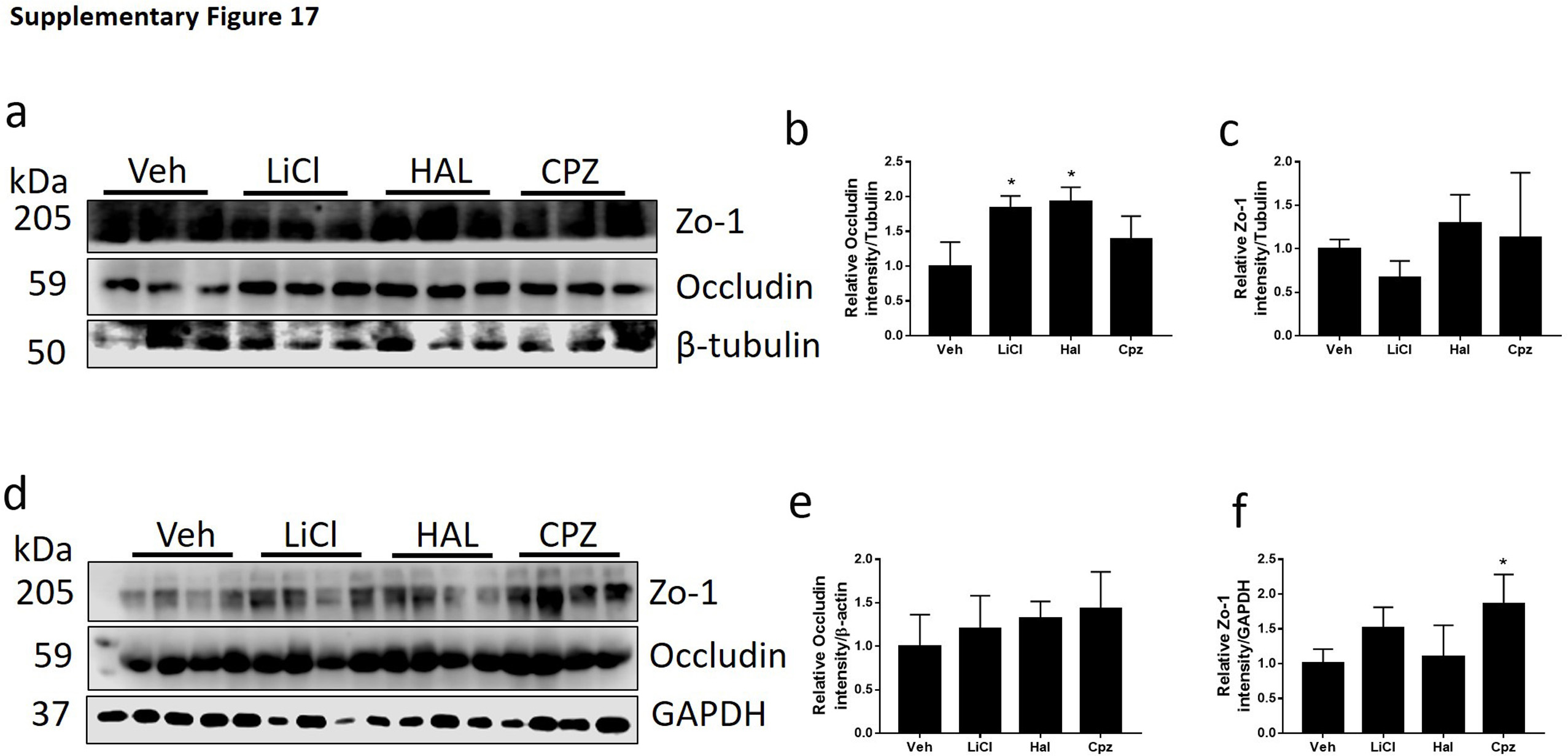

Supplement: Supplementary file 18 — Supplementary Figure 17 [file 41380_2018_149_MOESM18_ESM.jpg]

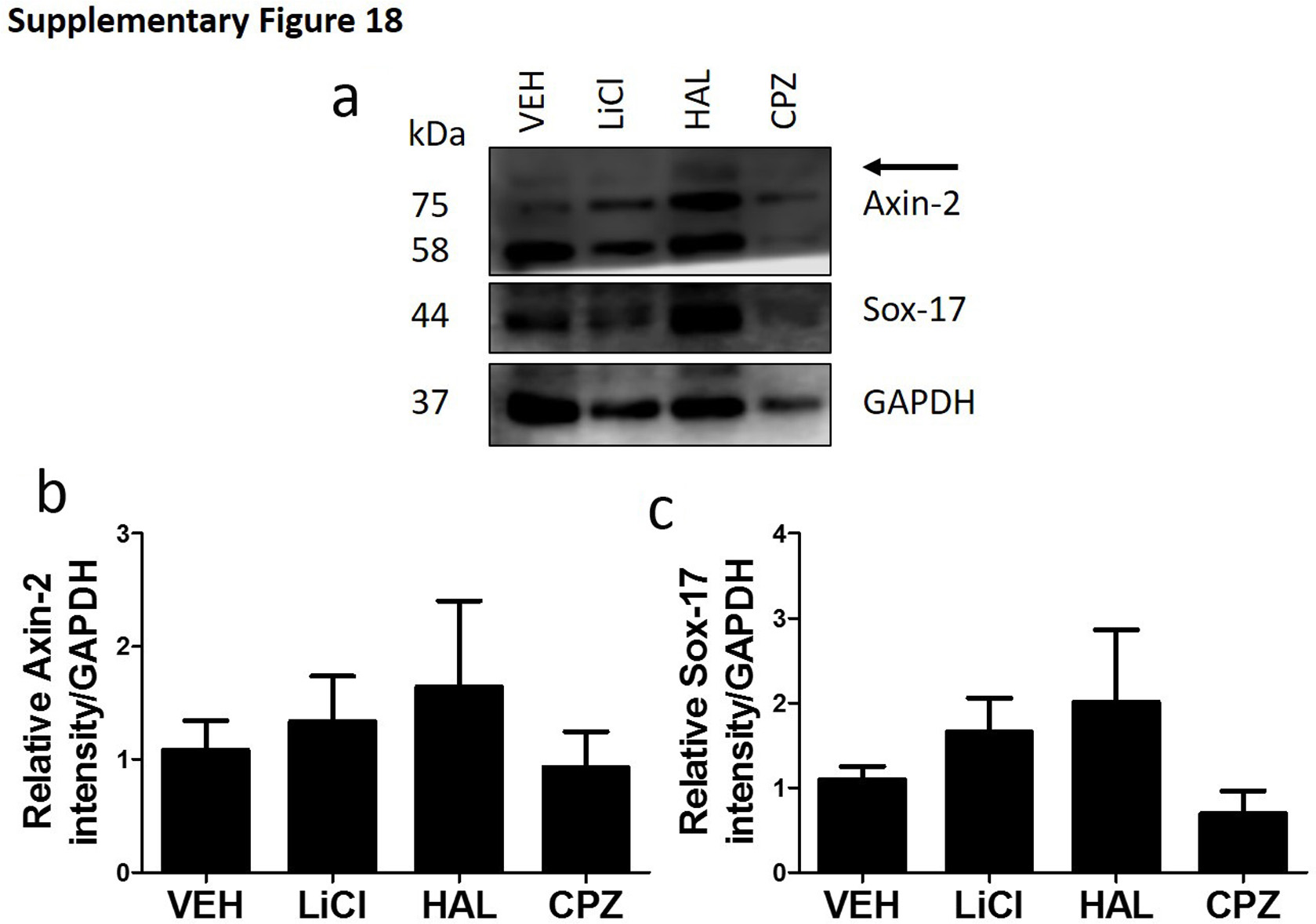

Supplement: Supplementary file 19 — Supplementary Figure 18 [file 41380_2018_149_MOESM19_ESM.jpg]

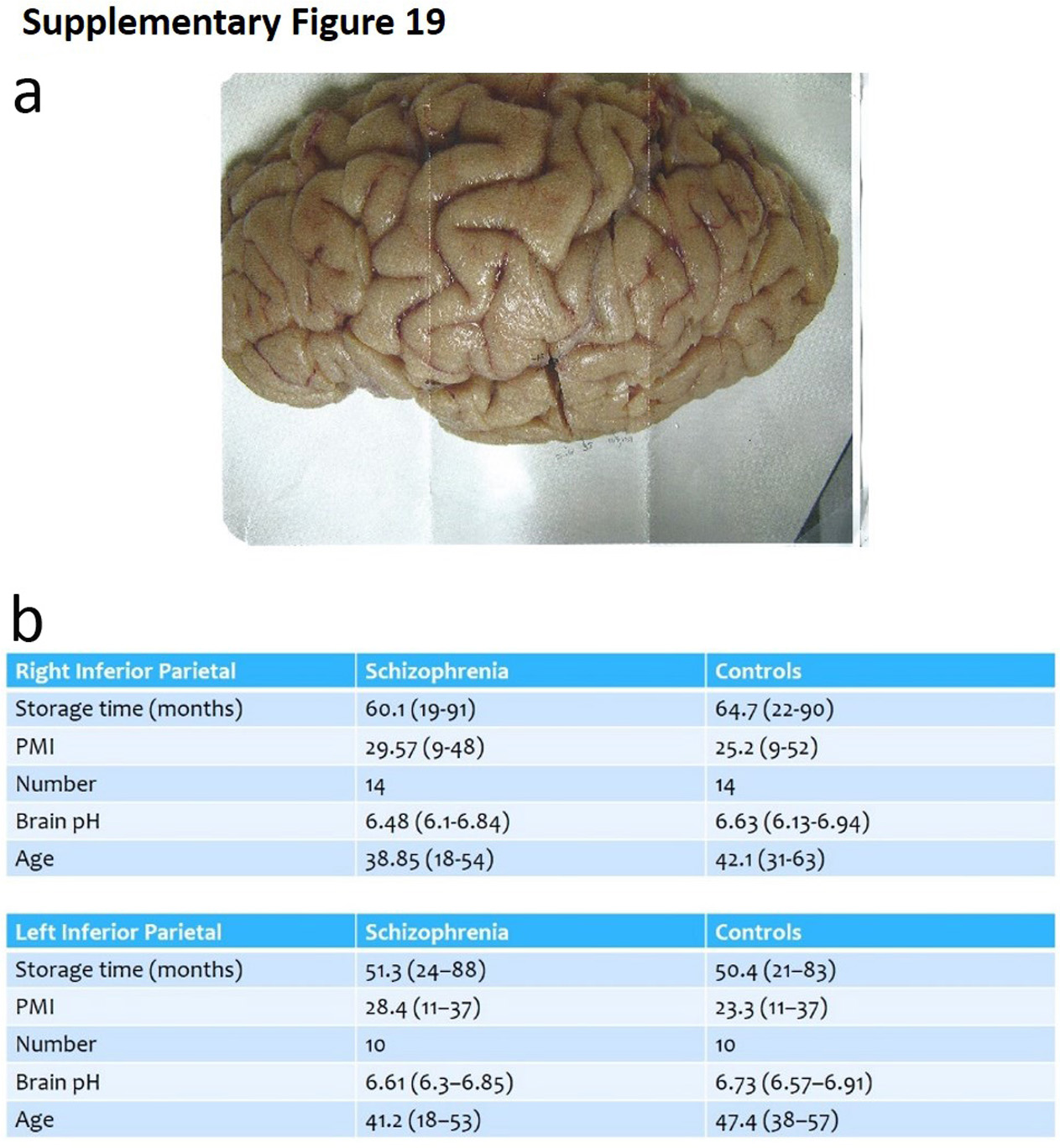

Supplement: Supplementary file 20 — Supplementary Figure 19 [file 41380_2018_149_MOESM20_ESM.jpg]
