# Supplementary material for: Dose-dependent expression of claudin-5 is a modifying factor in schizophrenia
Source: Mol Psychiatry. 2017 Oct 10;23(11):2156–66. doi: 10.1038/mp.2017.156 (PMC6298981; doi:10.1038/mp.2017.156)
Supplement: Supplementary file 21 — Supplementary Table 1 [file 41380_2018_149_MOESM21_ESM.pdf]

Geographical Region	Geographical Sub-Regions	Primary Language Family
**Arctic/Siberia/Central Asia**	Eastern Siberia and Greenland	Turkic<br>Chukchi–Kamchatkan<br><br>Eskimo–Aleut<br><br>Nivkh<br>Tungusic
	South-Central Siberia	Mongolic<br>Turkic
	Central Asia	Indo-European<br>Turkic
	Central/Western Siberia	Uralic<br>Uralic<br>Yeniseian
**Southeast and East Asia**	East Asia	Japonic<br>Koreanic<br>Tungusic<br>Sino-Tibetan<br>Hmong-Mien<br>Mongolic
	Mainland Southeast Asia	Tai-Kadai<br>Austroasiatic<br>Sino-Tibetan
**Americas**	Andes and Gran Chaco	Arawak<br>Matacoan<br>Quechuan
	Mesoamerica	Mayan<br>Mixe–Zoque<br>Oto-Manguean
	North America	Athabascan<br>Uto-Aztecan
	Amazon	Tupian<br>Arawak
**Australasia/Oceania/Island Southeast Asia**	Australia	Australian Languages *
	Island Southeast Asia	Austronesian
	Papua New Guinea	Papuan Languages *<br>Bougainville Languages *
	Polynesia	Austronesian
**South Asia**	Nepal	Indo-European<br>Kusunda

	Pakistan	Indo-European<br><br>Dravidian<br>Burushaski<br>-
	India and Bangladesh	Dravidian<br><br><br>Indo-European<br>Austroasiatic
**Africa**	Central Africa	-
	East Africa	Afro-Asiatic<br>Nilo-Saharan<br>Niger-Congo
	North Africa	Afro-Asiatic
	West Africa	Niger-Congo<br>Niger-Congo<br>-
	South Africa	Niger-Congo<br>Khoe/Kx'a
**Europe**	Volga/Ural	Uralic<br><br><br>Turkic
	Northeast Europe	Uralic<br><br>Indo-European
	Eastern Europe	Indo-European<br><br><br>Uralic
	Southern Europe	Indo-European
	Western Europe	Indo-European<br><br>Basque
**Western Asia**	South Caucasus and Turkey	Kartvelian<br>Indo-European<br>Northeast Caucasian<br>Turkic
	Iran	Indo-European
	North Caucasus	Northwest Caucasian<br>Turkic<br>Northeast Caucasian<br>Indo-European
	Arabian Peninsula, the Levant and Mesopotamia	Afro-Asiatic<br>Afro-Asiatic

	Afro-Asiatic

Language Grouping(s)	Population	Population No. Individuals
North Siberian	Yakut	12
Chuckchi	Chuckchi	6
Koryak	Koryak	16
Itelmen	Itelmen	1
Yukip-Inuit	Eskimo/Greenland Inuit	11
Aleut	Aleut	2
-	Nivkh	2
Northern Tungusic	Even/Evenk/Oroqen	26
Southern Tungusic	Ulchi/Hezhen	4
-	Buryat	17
South Siberian	Altaian/Shor/Tubalar/Telengit/Tuvan	17
Iranian	Tajik/Shugnan/Rushan-Vanch/Yaghnobi	9
Northwestern	Kazakh/Kyrgyz	12
Southeastern	Uygur/Uzbek	6
Southwestern	Turkmen	3
Samoyedic	Nganasan/Selkup/Nenets	11
Mansi/Khanty	Mansis/Khanty	8
Ket	Ket	3
Japanese	Japanese	3
Korean	Korean	2
Southern Tungusic	Xibo	2
Chinese/Tujia	Han/Tujia	6
Hmongic	She/Miao	4
-	Mongolian/Daur/Tu	11
Tai	Thai/Dai	7
Khmer/Vietic	Cambodian/Vietnamese	14
Lolo-Burmese/Qiangic	Burmese/Yi/Naxi	15
Tibetic	Lahu	2
Bolivia–Parana	Chané	1
-	Wichi	4
-	Cachi/Quecha/Kolla	12
-	Mayan	2
-	Mixe	3
-	Mixtec/Zapotec	4
-	Athabascan	2
-	Pima	2
-	Karitiana/Surui	6
Upper Amazon	Piapoco	2
	Australian	2
Malayo-Polynesian (Phillippine)	Batak/Vizayan/Igorot/Luzon	17
Adopted Malayo-Polynesian	Aeta/Agta	6
Malayo-Polynesian (Bornean)	Bajo/Dusun/Lebbo/Murut	26
Formosan	Ami/Atayal	3
	Papuan	22
	Bougainville	2
Polynesian	Maori/Hawaiian	2
Indo-Aryan	Tamang/Brahmin	2
-	Kusunda	2

Indo-Aryan	Sindhi/Punjabi/Kalash	8
Iranian	Pathan/Balochi/Hazara	6
Northern	Brahui	2
	Burusho	2
-	Makrani	2
Southern/South-Central	Balija/Gond/Kapu/Madiga/Mala/Irula/Malayan/Konda Dora	17
Indo-Aryan	Brahmin/Gupta/Kshatriya/Kol/Kurmi/Punjab/Thakur/Bengali/Orissa/Reli/Dhaka-mixed-pop/Madhya-Pradesh/Marwadi	20
Munda	Asur/Ho/Santhal	3
-	Central African Pygmies	9
Cushtic	Somali	1
Nilotic	Dinka/Luo/Masai	8
Northeast Bantu	BantuKenya/Luhya	4
Berber/Arab	Mozabite/Saharawi	4
Volta-Niger	Esan/Yoruba	6
Mande	Mandenka/Mende	6
-	Gambian	2
Southwest Bantu/Southern Bantu	BantuBotswana/Namibia	4
-	Ju hoan North/Khomani San	7
Permic	Komis/Udmurd	6
Mari	Mari	4
Mordvinic	Mordvins	3
Northwestern	Tatar/Bashkir	12
Southwestern	Chuvash	3
Finnic	Estonian/Finnish/Karelian/Vepsian/Ingrian	24
Sami	Sami	5
Baltic	Lithuanian/Latvian	6
West Slavic	Pole/Czech	6
East Slavic	Cossack/Russian/Ukrainian/Belarusian	24
Latin	Moldovan	2
Hungarian	Hungarian	4
Latin	Tuscan/Bergamo/Sardinian	8
South Slavic	Croat/Bulgarian	6
Greek	Cretan/Greek	4
Albanian	Albanian	4
Indo-Aryan	Romani	3
Germanic	English/Norwegian/Swedish/German/Orcadian/Icelandic	12
Latin	Spanish/French	5
	Basque	2
Georgian	Georgian	4
Armenian	Armenian	8
Abazgi	Abkhazian	5
Southwestern	Azerbaijani/Turkish	5
Iranian	Iranian	6
Circassian	Adygei/Circassian/Kabardin	9
Northwestern	Balkar/Kumyk	6
Avar-Andic/Lezgic/Nakh	Avar/Chechen/Lezgin/Tabasaran	13
Iranian	North Ossetian	4
Arabic	Arab/Bedouin/Druze/Jordanian/Palestinian/Saudi	23
Hebrew/Arabic	Samaritan/Iraqi Jew/Yemenite Jew	5

Neo-Aramaic	Assyrian	3

Population Derived Allele Frequency	Sub-Region No. Individuals	Sub-Region Derived Allele Frequency	Region No. Individuals	Region Derived Allele Frequency
66.67%	80	39.38%	166	33.13%
0.00%				
53.13%				
-				
27.27%				
0.00%				
25.00%				
42.31%				
12.50%				
32.35%	34	33.82%		
35.29%				
16.67%	30	26.67%		
20.83%				
33.33%				
66.67%				
18.18%	22	18.18%		
6.25%				
50.00%				
0.00%	28	33.93%	66	31.06%
25.00%				
75.00%				
33.33%				
50.00%				
31.82%				
35.71%	38	28.95%		
35.71%				
16.67%				
50.00%				
-	17	41.18%	38	30.26%
37.50%				
45.83%				
0.00%	9	27.78%		
50.00%				
25.00%				
50.00%	4	25.00%		
0.00%				
16.67%	8	12.50%		
0.00%				
25.00%	2	25.00%	80	17.50%
13.04%	52	18.27%		
8.33%				
23.08%				
16.67%				
18.18%	24	16.67%		
0.00%				
0.00%	2	0.00%		
50.00%	4	37.50%	64	14.84%
25.00%				

12.50%	20	17.50%		
25.00%				
0.00%				
50.00%				
0.00%				
8.82%	40	11.25%		
12.50%				
16.67%				
22.20%	9	22.20%	51	14.70%
0.00%	13	19.23%		
25.00%				
12.50%				
12.50%	4	12.50%		
8.30%	14	10.71%		
16.70%				
0.00%				
12.50%	11	9.09%		
7.10%				
16.67%	28	25.00%	143	14.34%
12.50%				
33.33%				
33.33%				
16.67%				
16.67%	35	15.71%		
10.00%				
16.67%				
33.33%	36	12.50%		
8.33%				
25.00%				
0.00%				
6.25%	25	10.00%		
8.33%				
12.50%				
12.50%				
16.67%				
4.17%	19	5.26%		
0.00%				
25.00%				
25.00%	22	20.00%	91	10.99%
25.00%				
20.00%				
10.00%				
16.67%	6	16.67%		
16.67%	32	10.94%		
8.33%				
11.54%				
0.00%				
2.17%	31	3.23%		
0.00%				

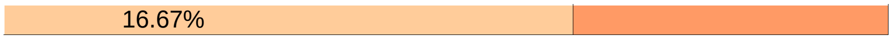
16.67%
