# Supplementary material for: Dose-dependent expression of claudin-5 is a modifying factor in schizophrenia
Source: Mol Psychiatry. 2017 Oct 10;23(11):2156–66. doi: 10.1038/mp.2017.156 (PMC6298981; doi:10.1038/mp.2017.156)
Supplement: Supplementary file 25 — Supplementary Movie Legends [file 41380_2018_149_MOESM25_ESM.docx]

Supplementary Video 1:  Seizure like activity and hyper locomotion behaviour was evident in mice post suppression of claudin-5 and within 24-48 hours prior to death.

Supplementary Video 2:  A distinct tail flickering phenotype was evident in mice post suppression of claudin-5 and within 48 hours prior to death.
